# Publisher Correction: scCASE: accurate and interpretable enhancement for single-cell chromatin accessibility sequencing data

**DOI:** 10.1038/s41467-024-46563-7

**Published:** 2024-03-12

**Authors:** Songming Tang, Xuejian Cui, Rongxiang Wang, Sijie Li, Siyu Li, Xin Huang, Shengquan Chen

**Affiliations:** 1 School of Mathematical Sciences and LPMC, Nankai University, Tianjin, 300071 China; 2 MOE Key Laboratory of Bioinformatics and Bioinformatics Division of BNRIST, Department of Automation, Tsinghua University, 100084 Beijing, China; 3 Department of Computer Science, University of Virginia, Charlottesville, VA 22903 USA; 4 School of Statistics and Data Science, Nankai University, Tianjin, 300071 China; 5 Beijing Key Laboratory for Radiobiology, Department of Radiation Biology, Beijing Institute of Radiation Medicine, 100850 Beijing, China

**Keywords:** Computational models, Data mining, Machine learning, DNA sequencing, Data processing

Correction to: *Nature Communications* 10.1038/s41467-024-46045-w, published online 22 February 2024

In this article the legend for Fig. 6, had the text “PLEASEKEYTHETEXTHERE” inadvertently inserted into it. The original article has been corrected.

