# Supplementary material for: scCASE: accurate and interpretable enhancement for single-cell chromatin accessibility sequencing data
Source: Nat Commun. 2024 Feb 22;15:1629. doi: 10.1038/s41467-024-46045-w (PMC10884038; doi:10.1038/s41467-024-46045-w)
Supplement: Supplementary file 1 — Supplementary Information [file 41467_2024_46045_MOESM1_ESM.pdf]

Supplementary Information for

scCASE: Accurate and interpretable enhancement for single-cell chromatin accessibility sequencing data

Songming Tang[1], Xuejian Cui[2], Rongxiang Wang[3], Sijie Li[1], Siyu Li[4], Xin Huang[5] & Shengquan Chen[1, *]

[1] School of Mathematical Sciences and LPMC, Nankai University, Tianjin 300071, China.

[2] MOE Key Laboratory of Bioinformatics and Bioinformatics Division of BNRIST, Department of Automation, Tsinghua University, Beijing 100084, China.

[3] Department of Computer Science, University of Virginia, Charlottesville, VA 22903, USA.

[4] School of Statistics and Data Science, Nankai University, Tianjin 300071, China.

[5] Beijing Key Laboratory for Radiobiology, Department of Radiation Biology, Beijing Institute of Radiation Medicine, Beijing 100850, China.

* Corresponding author: chenshengquan@nankai.edu.cn

Contents

Supplementary Texts **4**
- **Supplementary Text S1** **4**
- **Supplementary Text S2** **7**
- **Supplementary Text S3** **11**
- **Supplementary Text S4** **14**
- **Supplementary Text S5** **18**
- **Supplementary Text S6** **19**
- **Supplementary Text S7** **21**

Supplementary Figures **24**
- **Supplementary Figure S1** **24**
- **Supplementary Figure S2** **25**
- **Supplementary Figure S3** **26**
- **Supplementary Figure S4** **27**
- **Supplementary Figure S5** **28**
- **Supplementary Figure S6** **29**
- **Supplementary Figure S7** **30**
- **Supplementary Figure S8** **31**
- **Supplementary Figure S9** **32**
- **Supplementary Figure S10** **33**
- **Supplementary Figure S11** **34**
- **Supplementary Figure S12** **35**
- **Supplementary Figure S13** **36**
- **Supplementary Figure S14** **37**
- **Supplementary Figure S15** **38**
- **Supplementary Figure S16** **39**
- **Supplementary Figure S17** **40**
- **Supplementary Figure S18** **41**
- **Supplementary Figure S19** **42**

Supplementary Figure S20 43
Supplementary Figure S21 44
Supplementary Figure S22 45
Supplementary Figure S23 46
Supplementary Figure S24 47
Supplementary Figure S25 48
Supplementary Figure S26 49
Supplementary Figure S27 50
Supplementary Figure S28 51
Supplementary Figure S29 52
Supplementary Figure S30 53
Supplementary Tables 54
Supplementary Table S1 54
Supplementary Table S2 55
Supplementary Table S3 57
Supplementary Table S4 58
Supplementary Table S5 59
Supplementary Table S6 60
Supplementary References 62

Supplementary Texts

Supplementary Text S1: Details of the evaluation metrics.

For numerical accuracy, the precision recall curve (PRC) computes precision-recall pairs for different probability thresholds. The precision is the ratio $\frac{tp}{tp + fp}$ where tp is the number of true positives and fp the number of false positives. The recall is the ratio $\frac{tp}{tp + fn}$ where tp is the number of true positives and fn the number of false negatives. The last precision and recall values are 1 and 0 respectively and do not have a corresponding threshold. A receiver operating characteristic (ROC) is a graphical plot that illustrates the performance of a binary classifier system as its discrimination threshold is varied. It is created by plotting the fraction of true positives out of the positives (true positive rate, TPR) vs. the fraction of false positives out of the negatives (false positive rate, FPR), at various threshold settings. TPR is also known as sensitivity, and FPR is one minus the specificity or true negative rate.

For ARI, suppose that C are the actual labels of the clusters and K are the clustering labels. In that case, we define a and b as follows: a, the number of pairs of elements in the same set in C and the same set in K. b, the number of pairs of elements in different sets in C and different sets in K. The following formula then gives the original Rand index:

$$RI = \frac{a + b}{C_2^{n_{\text{samples}}}} \quad (1)$$

$C_2^{n_{\text{samples}}}$ is the total number of possible pairs in the datasets, and the following formula gives the ARI, which ensures random matching will get a value close to 0 and perfect matching gets a value close to 1:

$$ARI = \frac{RI - E[RI]}{\max(RI) - E[RI]} \quad (2)$$

Mutual Information (MI) evaluates the degree of dependence between two random variables. AMI adjusts MI by considering the expected value under random clustering, as shown in Equation (3).

$$AMI = \frac{MI(\mathbf{P}, \mathbf{T}) - E[MI(\mathbf{P}, \mathbf{T})]}{avg[H(\mathbf{P}), H(\mathbf{T})] - E[MI(\mathbf{P}, \mathbf{T})]} \quad (3)$$

Besides, the Fowlkes-Mallows Index (FMI) can be used as the geometric mean of pairwise precision and recall.

$$FMI = \frac{TP}{\sqrt{(TP + FP)(TP + FN)}} \quad (4)$$

The silhouette score evaluates the quality of clustering based on the distance between and within clusters. It is calculated using the Equation (5), where x_i represents the ith cell (totally N cells), and $a(x_i)$ and $b(x_i)$ represent the average distances between x_i and cells within and outside its cluster, respectively.

$$Silhouette\ score = \frac{1}{N} \sum_{i=0}^{N} \frac{b(x_i) - a(x_i)}{\max\big(a(x_i), b(x_i)\big)} \quad (5)$$

While silhouette score is commonly utilized to evaluate clustering results, we replaced the cluster labels with the cell type labels to evaluate the performance of the impact of enhancement on the estimation of the distance between cells, and the higher the silhouette score, the better the performance.

k-nearest neighbour batch effect test (kBET) is a wrapper function of the implementation by Büttner et al.[1]. kBET measures the bias of a batch variable in the kNN graph. Specifically, kBET is quantified as the average rejection rate of Chi-squared tests of local vs global batch label distributions. This means that smaller values indicate better batch mixing. Batch ASW measures the silhouette of a given batch[2]. It assumes that a silhouette width close to 0 represents

a perfect overlap of the batches, thus the absolute value of the silhouette width is used to measure how well batches are mixed. We calculated kBET and Batch ASW using scib[2] and by default, the original kBET score and Batch ASW are scaled between 0 and 1 so that larger scores are associated with better batch mixing.

For over-smoothing score, under-smoothing score, and smoothing score, we posit that the data affected by over-smoothing would be an exaggerated similarity between certain distinct cell types. In contrast, well-smoothed data would lead to closer distances between cells of the same type, whereas distances between a cell and another cell from different types would be greater than those between two cells of the same type. Based on this, let $d(x1, x2)$ represent the distance between cells x_1 and x_2 (assumed to be the Euclidean distance in the original dimensions in this study). For a given cell x, let a be the average distance between x and other cells of the same type with x, and let b be the average distance between x and cells of the nearest different cell type. We can compute the averages $\bar{a}$ and $\bar{b}$ for a and b of all cells in a dataset, and calculate the average distance $\bar{d}$ between any two cells in the dataset. We define the over-smoothing score as $\frac{\bar{b}}{\bar{d}}$ and the under-smoothing score is $1-\frac{\bar{a}}{\bar{d}}$. For the over-smoothing score, a low score implies that each cell has a close distance to a different cell type, indicating excessive smoothing that eliminates cellular heterogeneity. Regarding the under-smoothing score, a lower value suggests that the distances between cells of the same type have not become closer after enhancement. We compute the harmonic mean of the over-smoothing score and under-smoothing score, referred to as the smoothing score.

$$\text{smoothing score} = \frac{2 \times \text{over smoothing score} \times \text{under smoothing score}}{\text{over smoothing score} + \text{under smoothing score}} \quad (6)$$

Supplementary Text S2: Details of scCASE with reference data.

To harness large compendia of available omics data and improve the performance of downstream analyses, we have expanded upon the scCASE method and developed scCASER (scCASE with reference data). By incorporating reference data, scCASER enables more accurate enhancement of scCAS data. Given scCAS count matrix $\mathbf{X} \in \mathbb{R}^{m\times n}$, where x_{ij} is the read count in peak i and cell j, m is the number of peaks, and n is the total number of cells. The optimization problem of scCASER is as follows.

$$\begin{gathered}\min_{\mathbf{W_{1,2}},\mathbf{H},\mathbf{Z}\geq 0} F = \| \mathbf{X}(\mathbf{Z}\circ\mathbf{R}) - [\mathbf{W_1},\mathbf{W_2}]\mathbf{H} \|_F^2 + \lambda \| \mathbf{Z} - \mathbf{H}^T\mathbf{H} \|_F^2 + \\ \gamma_1 \| \mathbf{W_m} \|_F^2 + \gamma_2 \| \mathbf{H} \|_F^2 + \alpha \| \mathbf{P} - \mathbf{W_1} \|_F^2\end{gathered} \tag{7}$$

which equals to:

$$\begin{gathered}\min_{\mathbf{W_{1,2}},\mathbf{H},\mathbf{Z}\geq 0} F = \| \mathbf{X}(\mathbf{Z}\circ\mathbf{R}) - (\mathbf{M}\circ\mathbf{W_1} + \mathbf{N}\circ\mathbf{W_2})\mathbf{H} \|_\mathrm{F}^2 + \lambda \| \mathbf{Z} - \mathbf{H}^T\mathbf{H} \|_\mathrm{F}^2 + \\ \gamma_1 \| \mathbf{W_m} \|_\mathrm{F}^2 + \gamma_2 \| \mathbf{H} \|_\mathrm{F}^2 + \alpha \| \mathbf{P} - \mathbf{W_1} \|_\mathrm{F}^2\end{gathered} \tag{8}$$

$\mathbf{Z} \in \mathbb{R}^{n\times n}$ is the similarity matrix, $\mathbf{R} \in \mathbb{R}^{n\times n}$ is a random sample matrix. $\mathbf{H} \in \mathbb{R}^{k\times n}$ is the cell embedding matrix of scCAS data, $\mathbf{W_m} \in \mathbb{R}^{m\times k}$ is the projection matrix and can be written as $[\mathbf{W_1},\mathbf{W_2}]$, where $\mathbf{W_1} \in \mathbb{R}^{m\times k_1}$ is the part obtained from the reference data, and $\mathbf{W_2} \in \mathbb{R}^{m\times k_2}$ is the part obtained from the target scCAS data, with $k = k_1 + k_2$. $\mathbf{P} \in \mathbb{R}^{m\times k}$ is the projection matrix obtained from reference data using NMF. $\mathbf{M}$, $\mathbf{N}$ are mask matrices, making the $\mathbf{W_1}$, $\mathbf{W_2}$ work on specific dimensions. We let $\mathbf{W_m} = \mathbf{M}\circ\mathbf{W_1} + \mathbf{N}\circ\mathbf{W_2}$, and the loss function F can also be written as below:

$$\begin{gathered}F = tr((\mathbf{Z}\circ\mathbf{R})^T\mathbf{X}^T\mathbf{X}(\mathbf{Z}\circ\mathbf{R}) - \mathbf{H}^T(\mathbf{M}\circ\mathbf{W_1} + \mathbf{N}\circ\mathbf{W_2})^T\mathbf{X}(\mathbf{Z}\circ\mathbf{R}) - (\mathbf{Z}\circ\mathbf{R})^T\mathbf{X}^T(\mathbf{M}\circ\mathbf{W_1} + \mathbf{N}\circ\mathbf{W_2})\mathbf{H}) \\ +tr(\mathbf{H}^T(\mathbf{M}\circ\mathbf{W_1} + \mathbf{N}\circ\mathbf{W_2})^\mathrm{T}(\mathbf{M}\circ\mathbf{W_1} + \mathbf{N}\circ\mathbf{W_2})\mathbf{H}) \\ +\lambda tr(\mathbf{Z}^T\mathbf{Z} - \mathbf{H}^T\mathbf{H}\mathbf{Z} - \mathbf{Z}^\mathrm{T}\mathbf{H}^\mathrm{T}\mathbf{H} + \mathbf{H}^\mathrm{T}\mathbf{H}\mathbf{H}^\mathrm{T}\mathbf{H}) \\ +\gamma_1 tr(\mathbf{W_m}^T\mathbf{W_m}) + \gamma_2 tr(\mathbf{H}^T\mathbf{H}) \\ +\alpha tr\left(\mathbf{P}^T\mathbf{P} - (\mathbf{M}\circ\mathbf{W_1})^T\mathbf{P} - \mathbf{P}^T(\mathbf{M}\circ\mathbf{W_1}) + (\mathbf{M}\circ\mathbf{W_1})^T(\mathbf{M}\circ\mathbf{W_1})\right)\end{gathered} \tag{9}$$

The partial derivatives of F with respect to $\mathbf{W_1}$, $\mathbf{W_2}$, $\mathbf{H}$ and $\mathbf{Z}$ are shown in Equation (10).

$$
\begin{aligned}
\frac{\partial F}{\partial \mathbf{W_1}} &= (-2\mathbf{X}(\mathbf{Z}\circ\mathbf{R})\mathbf{H}^{\mathrm{T}} + 2\mathbf{W_m}\mathbf{H}\mathbf{H}^{T} + 2\mathbf{W_m} - \mathbf{2}\alpha\mathbf{P} + \mathbf{2}\alpha\mathbf{M}\circ\mathbf{W_1})\circ\mathbf{M} \\
\frac{\partial F}{\partial \mathbf{W_2}} &= (-2\mathbf{X}(\mathbf{Z}\circ\mathbf{R})\mathbf{H}^{\mathrm{T}} + 2\mathbf{W_m}\mathbf{H}\mathbf{H}^{T} + 2\mathbf{W_m})\circ\mathbf{N} \\
\frac{\partial F}{\partial \mathbf{H}} &= -2\mathbf{W}_\mathbf{m}^{T}\mathbf{X}(\mathbf{Z}\circ\mathbf{R}) + 2\mathbf{W}_\mathbf{m}^{T}\mathbf{W_m}\mathbf{H} - 2\lambda\mathbf{H}(\mathbf{Z}+\mathbf{Z}^{T}) + 4\lambda\mathbf{H}\mathbf{H}^{T}\mathbf{H} + 2\gamma_2\mathbf{H} \\
\frac{\partial F}{\partial \mathbf{Z}} &= 2(\mathbf{X}^{T}\mathbf{X}(\mathbf{Z}\circ\mathbf{R}))\circ\mathbf{R} - 2\mathbf{X}^{T}\mathbf{W_m}\mathbf{H}\circ\mathbf{R} + 2\lambda\mathbf{Z} - 2\lambda\mathbf{H}^{T}\mathbf{H}
\end{aligned}
\tag{10}
$$

We use the gradient descent method to update these matrices, and we choose the optimal step size for each iteration to improve the efficiency of the optimization algorithm. Let $\frac{\partial F}{\partial \mathbf{W_1}} = \mathbf{D_1}\circ\mathbf{M}$, $\frac{\partial F}{\partial \mathbf{W_2}} = \mathbf{D_2}\circ\mathbf{N}$, $\frac{\partial F}{\partial \mathbf{Z}} = \mathbf{D_3}$, $\frac{\partial F}{\partial \mathbf{H}} = \mathbf{D_4}$. For $\mathbf{W_1}$, suppose the optimal step length is δ, then the δ will let $F(\mathbf{W_1} - \delta\mathbf{D_1}\circ\mathbf{M}, \mathbf{W_2}, \mathbf{H}, \mathbf{Z})$ get minimum. In this case, we have $\frac{dF(\mathbf{W_1}-\delta\mathbf{D_1}\circ\mathbf{M},\mathbf{W_2},\mathbf{H},\mathbf{Z})}{d\delta} = 0$. The calculation of loss function F is shown below.

$$
\begin{gathered}
F = tr((\mathbf{Z}\circ\mathbf{R})^{T}\mathbf{X}^{T}\mathbf{X}(\mathbf{Z}\circ\mathbf{R}) - \mathbf{H}^{T}(\mathbf{M}\circ(\mathbf{W_1}-\delta\mathbf{D_1}) + \mathbf{N}\circ\mathbf{W_2})^{T}\mathbf{X}(\mathbf{Z}\circ\mathbf{R}) \\
-(\mathbf{Z}\circ\mathbf{R})^{T}\mathbf{X}^{T}(\mathbf{M}\circ(\mathbf{W_1}-\boldsymbol{\delta}\mathbf{D_1}) + \mathbf{N}\circ\mathbf{W_2})\mathbf{H} \\
+\mathbf{H}^{T}(\mathbf{M}\circ(\mathbf{W_1}-\delta\mathbf{D_1}) + \mathbf{N}\circ\mathbf{W_2})^{T}(\mathbf{M}\circ(\mathbf{W_1}-\delta\mathbf{D_1}) + \mathbf{N}\circ\mathbf{W_2})\mathbf{H}) \\
+\boldsymbol{\lambda}\, tr(\mathbf{Z}^{T}\mathbf{Z} - \mathbf{H}^{T}\mathbf{H}\mathbf{Z} - \mathbf{Z}^{T}\mathbf{H}^{T}\mathbf{H} + \mathbf{H}^{T}\mathbf{H}\mathbf{H}^{T}\mathbf{H}) \\
+\gamma_1\, tr(\mathbf{W}_\mathbf{m}^{T}\mathbf{W_m}) + \gamma_2\, tr(\mathbf{H}^{T}\mathbf{H}) + \alpha\, tr\Big(\mathbf{P}^{T}\mathbf{P} - \big(\mathbf{M}\circ(\mathbf{W_1}-\delta\mathbf{D_1})\big)^{T}\mathbf{P} \\
-\mathbf{P}^{T}\big(\mathbf{M}\circ(\mathbf{W_1}-\delta\mathbf{D_1})\big) + \big(\mathbf{M}\circ(\mathbf{W_1}-\delta\mathbf{D_1})\big)^{T}\big(\mathbf{M}\circ(\mathbf{W_1}-\delta\mathbf{D_1})\big)\Big)
\end{gathered}
\tag{11}
$$

$$
\begin{gathered}
\frac{dF}{d\delta} = 0 = tr\left(\mathbf{M}\circ\mathbf{H}^{T}\mathbf{D}_\mathbf{1}^{T}\mathbf{X}(\mathbf{Z}\circ\mathbf{R})\right) + tr(\mathbf{M}\circ(\mathbf{Z}\circ\mathbf{R})^{T}\mathbf{X}^{T}\boldsymbol{D_1}\boldsymbol{H}) \\
-\, tr\, \mathbf{H}^{T}\big((\mathbf{N}\circ\mathbf{W_2})^{T}(\mathbf{M}\circ\mathbf{D_1}) + (\mathbf{M}\circ\mathbf{D_1})^{T}(\mathbf{N}\circ\mathbf{W_2})\big)\mathbf{H} \\
-\, tr(\mathbf{H}^{T}(\mathbf{M}\circ\mathbf{D_1})^{T}(\mathbf{M}\circ\mathbf{W_1}) + (\mathbf{M}\circ\mathbf{W_1})^{T}(\mathbf{M}\circ\mathbf{D_1})\mathbf{H}) \\
+2\delta\, tr(\mathbf{H}^{T}(\mathbf{M}\circ\mathbf{D_1})^{T}(\mathbf{M}\circ\mathbf{D_1})\mathbf{H}) \\
+\alpha\, tr(\mathbf{P}^{T}(\mathbf{M}\circ\mathbf{D_1}) - (\mathbf{M}\circ\mathbf{D_1})^{\mathrm{T}}\mathbf{P}) \\
-\alpha\, tr\big((\mathbf{M}\circ\mathbf{D_1})^{T}(\mathbf{M}\circ\mathbf{W_1}) + (\mathbf{M}\circ\mathbf{W_1})^{\mathrm{T}}(\mathbf{M}\circ\mathbf{D_1})\big) \\
+2\alpha\delta\, tr\big((\mathbf{M}\circ\mathbf{D_1})^{T}(\mathbf{M}\circ\mathbf{D_1})\big)
\end{gathered}
\tag{12}
$$

Then we get the optimal δ value as Equation (13)

$$
\delta = \frac{-tr(\mathbf{eq1}) - tr(\mathbf{eq2}) + tr(\mathbf{eq3}) + tr(\mathbf{eq4}) - \alpha\, tr(\mathbf{eq6}) + \alpha tr(\mathbf{eq7})}{2\, tr(\mathbf{eq5}) + 2\alpha\, tr(\mathbf{eq8})}
\tag{13}
$$

$$
\begin{aligned}
\mathbf{eq1} &= \mathbf{M}\circ\mathbf{H}^T\mathbf{D}_1^T\mathbf{X}(\mathbf{Z}\circ\mathbf{R})\\
\mathbf{eq2} &= \mathbf{M}\circ(\mathbf{Z}\circ\mathbf{R})^T\mathbf{X}^T\boldsymbol{D_1H}\\
\mathbf{eq3} &= \mathbf{H}^T((\mathbf{N}\circ\mathbf{W}_2)^T(\mathbf{M}\circ\mathbf{D}_1)+(\mathbf{M}\circ\mathbf{D}_1)^T(\mathbf{N}\circ\mathbf{W}_2))\mathbf{H}\\
\mathbf{eq4} &= \mathbf{H}^T((\mathbf{M}\circ\mathbf{D}_1)^T(\mathbf{M}\circ\mathbf{W}_1)+(\mathbf{M}\circ\mathbf{W}_1)^T(\mathbf{M}\circ\mathbf{D}_1))\mathbf{H}\\
\mathbf{eq5} &= \mathbf{H}^T((\mathbf{M}\circ\mathbf{D}_1)^T(\mathbf{M}\circ\mathbf{D}_1))\mathbf{H}\\
\mathbf{eq6} &= \mathbf{P}^T(\mathbf{M}\circ\mathbf{D}_1)-(\mathbf{M}\circ\mathbf{D}_1)^{\mathrm{T}}\mathbf{P}\\
\mathbf{eq7} &= (\mathbf{M}\circ\mathbf{D}_1)^T(\mathbf{M}\circ\mathbf{W}_1)+(\mathbf{M}\circ\mathbf{W}_1)^T(\mathbf{M}\circ\mathbf{D}_1)\\
\mathbf{eq8} &= (\mathbf{M}\circ\mathbf{D}_1)^T(\mathbf{M}\circ\mathbf{D}_1)
\end{aligned}
\tag{14}
$$

For $\mathbf{W_2}$, similarly, we suppose the optimal step length is δ, then δ will let $F(\mathbf{W_1},\mathbf{W_2}-\delta\mathbf{D_2}\circ\mathbf{N},\mathbf{H},\mathbf{Z})$ get minimum. In this case, we have $\frac{dF(\mathbf{W_1},\mathbf{W_2}-\delta\mathbf{D_2}\circ\mathbf{N},\mathbf{H},\mathbf{Z})}{d\delta}=0$. The calculation of loss function F is shown below.

$$
\begin{gathered}
F = tr\left((\mathbf{Z}\circ\mathbf{R})^T\mathbf{X}^T\mathbf{X}(\mathbf{Z}\circ\mathbf{R})-\mathbf{H}^T\big(\mathbf{M}\circ\mathbf{W_1}+\mathbf{N}\circ(\mathbf{W_2}-\delta\mathbf{D_2})\big)^T\mathbf{X}(\mathbf{Z}\circ\mathbf{R})\right.\\
-(\mathbf{Z}\circ\mathbf{R})^T\mathbf{X}^T\big(\mathbf{M}\circ\mathbf{W_1}+\mathbf{N}\circ(\mathbf{W_2}-\delta\mathbf{D_2})\big)\mathbf{H}\\
\left.+\mathbf{H}^T\big(\mathbf{M}\circ\mathbf{W_1}+\mathbf{N}\circ(\mathbf{W_2}-\delta\mathbf{D_2})\big)^T\big(\mathbf{M}\circ\mathbf{W_1}+\mathbf{N}\circ(\mathbf{W_2}-\delta\mathbf{D_2})\big)\mathbf{H}\right)\\
+\boldsymbol{\lambda}\,tr(\mathbf{Z}^T\mathbf{Z}-\mathbf{H}^T\mathbf{H}\mathbf{Z}-\mathbf{Z}^T\mathbf{H}^T\mathbf{H}+\mathbf{H}^T\mathbf{H}\mathbf{H}^T\mathbf{H})\\
+\gamma_1\,tr(\mathbf{W_m^T}\mathbf{W_m})+\gamma_2\,tr(\mathbf{H}^T\mathbf{H})+\alpha\,tr(\mathbf{P}^T\mathbf{P}-(\mathbf{M}\circ\mathbf{W_1})^T\mathbf{P}\\
-\mathbf{P}^T(\mathbf{M}\circ\mathbf{W_1})+(\mathbf{M}\circ\mathbf{W_1})^T(\mathbf{M}\circ\mathbf{W_1})\big)
\end{gathered}
\tag{15}
$$

$$
\begin{aligned}
\frac{dF}{d\delta}=\mathbf{0}= {} & tr\left(\mathbf{N}\circ\mathbf{H}^T\mathbf{D}_2^T\mathbf{X}(\mathbf{Z}\circ\mathbf{R})\right)+tr(\mathbf{N}\circ(\mathbf{Z}\circ\mathbf{R})^T\mathbf{X}^T\mathbf{D}_2\mathbf{H})\\
& -tr\big(\mathbf{H}^T\big((\mathbf{M}\circ\mathbf{W}_1)^T(\mathbf{N}\circ\mathbf{D}_2)+(\mathbf{N}\circ\mathbf{D}_2)^T(\mathbf{M}\circ\mathbf{W}_1)\big)\mathbf{H}\big)\\
& -tr\big(\mathbf{H}^T\big((\mathbf{N}\circ\mathbf{D}_2)^T(\mathbf{N}\circ\mathbf{W}_2)+(\mathbf{N}\circ\mathbf{W}_2)^T(\mathbf{N}\circ\mathbf{D}_2)\big)\mathbf{H}\big)\\
& +2\delta tr\big(\mathbf{H}^T\big((\mathbf{N}\circ\mathbf{D}_2)^T(\mathbf{N}\circ\mathbf{D}_2)\big)\mathbf{H}\big)
\end{aligned}
\tag{16}
$$

Then, we get the optimal δ value as Equation (17).

$$
\delta=\frac{-tr(\mathbf{eq1})-tr(\mathbf{eq2})+tr(\mathbf{eq3})+tr(\mathbf{eq4})}{2\,tr(\mathbf{eq5})}
\tag{17}
$$

$$
\begin{aligned}
\mathbf{eq1} &= \mathbf{N}\circ\mathbf{H}^T\mathbf{D}_2^T\mathbf{X}(\mathbf{Z}\circ\mathbf{R})\\
\mathbf{eq2} &= \mathbf{N}\circ(\mathbf{Z}\circ\mathbf{R})^T\mathbf{X}^T\mathbf{D}_2\mathbf{H}\\
\mathbf{eq3} &= \mathbf{H}^{\boldsymbol{T}}((\mathbf{M}\circ\mathbf{W}_1)^T(\mathbf{N}\circ\mathbf{D}_2)+(\mathbf{N}\circ\mathbf{D}_2)^T(\mathbf{M}\circ\mathbf{W}_1))\mathbf{H}\\
\mathbf{eq4} &= \mathbf{H}^T((\mathbf{N}\circ\mathbf{D}_2)^T(\mathbf{N}\circ\mathbf{W}_2)+(\mathbf{N}\circ\mathbf{W}_2)^T(\mathbf{N}\circ\mathbf{D}_2))\mathbf{H}\\
\mathbf{eq5} &= \mathbf{H}^T((\mathbf{N}\circ\mathbf{D}_2)^T(\mathbf{N}\circ\mathbf{D}_2))\mathbf{H}
\end{aligned}
\tag{18}
$$

For $\mathbf{Z}$, similarly, we suppose the optimal step length is δ, then δ will let $F(\mathbf{W_1},\mathbf{W_2},\mathbf{H},\mathbf{Z}-\boldsymbol{\delta}\mathbf{D_3})$ get minimum. In this case, we have $\frac{d\mathbf{F}(\mathbf{W_1},\mathbf{W_2},\mathbf{H},\mathbf{Z}-\boldsymbol{\delta}\mathbf{D_3})}{d\delta}=0$. The calculation of loss function F is shown as Equation (19).

$$
\begin{aligned}
F = tr(((\mathbf{Z}-\delta\mathbf{D_3})\circ\mathbf{R})^T\mathbf{X}^T\mathbf{X}(\mathbf{Z}-\delta\mathbf{D_3})\circ\mathbf{R} - \mathbf{H}^T(\mathbf{M}\circ\mathbf{W_1}+\mathbf{N}\circ\mathbf{W_2})^T\mathbf{X}(\mathbf{Z}-\delta\mathbf{D_3})\circ\mathbf{R} \\
-\big((\mathbf{Z}-\delta\mathbf{D_3})\circ\mathbf{R}\big)^T\mathbf{X}^T(\mathbf{M}\circ\mathbf{W_1}+\mathbf{N}\circ\mathbf{W_2})\mathbf{H} \\
+\mathbf{H}^T(\mathbf{M}\circ\mathbf{W_1}+\mathbf{N}\circ\mathbf{W_2})^T(\mathbf{M}\circ\mathbf{W_1}+\mathbf{N}\circ\mathbf{W_2})\mathbf{H}) \\
+\boldsymbol{\lambda}\, tr((\mathbf{Z}-\delta\mathbf{D_3})^T(\mathbf{Z}-\delta\mathbf{D_3}) - \mathbf{H}^T\mathbf{H}(\mathbf{Z}-\delta\mathbf{D_3}) - (\mathbf{Z}-\delta\mathbf{D_3})^T\mathbf{H}^T\mathbf{H} + \mathbf{H}^T\mathbf{H}\mathbf{H}^T\mathbf{H}) \\
+\gamma_1\, tr(\mathbf{W_m^T W_m}) + \gamma_2\, tr(\mathbf{H}^T\mathbf{H}) + \alpha\, tr(\mathbf{P}^T\mathbf{P} - (\mathbf{M}\circ\mathbf{W_1})^T\mathbf{P} \\
-\mathbf{P}^T(\mathbf{M}\circ\mathbf{W_1}) + (\mathbf{M}\circ\mathbf{W_1})^T(\mathbf{M}\circ\mathbf{W_1}))
\end{aligned} \tag{19}
$$

$$
\begin{aligned}
\frac{dF}{d\delta} = 0 = -\,tr((\mathbf{D_3}\circ\mathbf{R})^T\mathbf{X}^T\mathbf{X}(\mathbf{Z}\circ\mathbf{R}) + (\mathbf{Z}\circ\mathbf{R})^T\mathbf{X}^T\mathbf{X}(\mathbf{D_3}\circ\mathbf{R})) \\
+2\delta tr\big((\mathbf{D_3}\circ\mathbf{R})^T\mathbf{X}^T\mathbf{X}(\mathbf{D_3}\circ\mathbf{R})\big) \\
+tr(\mathbf{H}^T\mathbf{W_m^T}\mathbf{X}(\mathbf{D_3}\circ\mathbf{R}) + (\mathbf{D_3}\circ\mathbf{R})^T\mathbf{X}^T\mathbf{W_m}\mathbf{H}) \\
-\lambda tr(\mathbf{Z}^T\mathbf{D_3} + \mathbf{D_3^T}\mathbf{Z}) + 2\lambda\delta tr(\mathbf{D_3^T D_3}) \\
+\lambda tr(\mathbf{H}^T\mathbf{H}\mathbf{D_3} + \mathbf{D_3^T}\mathbf{H}^T\mathbf{H})
\end{aligned} \tag{20}
$$

Then, we get the optimal δ value as below.

$$
\delta = \frac{tr(\mathbf{eq1}) - tr(\mathbf{eq3}) + \lambda tr(\mathbf{eq4}) - \lambda\, tr(\mathbf{eq6})}{2\, tr(\mathbf{eq2}) + 2\lambda(\mathbf{eq5})} \tag{21}
$$

$$
\begin{aligned}
\mathbf{eq1} &= (\mathbf{D_3}\circ\mathbf{R})^T\mathbf{X}^T\mathbf{X}(\mathbf{Z}\circ\mathbf{R}) + (\mathbf{Z}\circ\mathbf{R})^T\mathbf{X}^T\mathbf{X}(\mathbf{D_3}\circ\mathbf{R})) \\
\mathbf{eq2} &= (\mathbf{D_3}\circ\mathbf{R})^T\mathbf{X}^T\mathbf{X}(\mathbf{D_3}\circ\mathbf{R}) \\
\mathbf{eq3} &= \mathbf{H}^T\mathbf{W_m^T}\mathbf{X}(\mathbf{D_3}\circ\mathbf{R}) + (\mathbf{D_3}\circ\mathbf{R})^T\mathbf{X}^T\mathbf{W_m}\mathbf{H} \\
\mathbf{eq4} &= \mathbf{Z}^T\mathbf{D_3} + \mathbf{D_3^T}\mathbf{Z} \\
\mathbf{eq5} &= \mathbf{D_3^T D_3} \\
\mathbf{eq6} &= \mathbf{H}^T\mathbf{H}\mathbf{D_3} + \mathbf{D_3^T}\mathbf{H}^T\mathbf{H}
\end{aligned} \tag{22}
$$

For $\mathbf{H}$, due to its high order terms, we cannot find the optimal step size in this way. We initialize the step size δ of $\mathbf{H}$ to 0.2. If this step size cannot reduce the loss function during the iteration process, we will reduce the step size and try updating $\mathbf{H}$ again.

Supplementary Text S3: Discussion and extension of scCASE for correcting sequencing depth.

Correcting for sequencing depth is one of the most important goals in denoising scCAS data since it is sparser than scRNA-seq, and regular transcripts per million (TPM) don't work. TF-IDF transformation is widely used to normalize the sequencing depth[3-5]. However, TF-IDF doesn't fully correct for sequencing depth. To assess the validity of scCASE in correcting for sequencing depth, following a workflow similar to Cusanovich DA et al.[6], we applied TF-IDF transformation to the raw count matrix and employed SVD to reduce the model dimensions to 10. We observed a significant correlation between PC1 and sequencing depth (Supplementary Figs. 7a, d; 8a, d). For instance, in the Blood dataset, the correlation coefficient between PC1 and sequencing depth exceeds 0.8. This is also evident in UMAP visualization, where sequencing depth largely determines the positions of cells in the low-dimensional representation (Supplementary Figs. S7, S8). This indicates that the raw data combined with TF-IDF does not effectively normalize the sequencing depth. In the LungA dataset, we observed similar results, the correlation coefficient between PC1 and sequencing depth exceeds 0.7 and the impact of sequencing depth on low-dimensional representation can be observed in UMAP visualization (Supplementary Figs. S7, S8). This indicates that TF-IDF does not effectively normalize the sequencing depth.

For the data enhanced by scCASE, we conducted a similar process, and the results indicate that, despite the absence of explicit modeling for sequencing depth, the observed correlation was somewhat attenuated. In the Blood dataset, the correlation coefficient between PC1 and sequencing depth has been reduced by 46.5%, while in the LungA dataset, it has been reduced

by 19.5% (Supplementary Figs. S7, S8). Given that scCASE takes into consideration the similarity between cells during modeling and similar cells generally exhibit comparable chromatin accessibility patterns, a particular cell, has numerous similar cells with varying sequencing depths. Representing read counts as the weighted average of multiple similar cells corrected the sequencing depth at the same time. Consequently, in the scCASE enhanced data, the influence of sequencing depth is mitigated to a certain extent.

To enhance the capability of scCASE in further mitigating the impact of sequencing depth, we extended the scCASE model and set it as optional for users. In this extended model, we explicitly incorporated sequencing depth for modeling by introducing the sequencing depth matrix $\mathbf{P} \in \mathbb{R}^{n \times n}$. The elements on the main diagonal of this matrix represent the sequencing depth of the cells. This matrix is utilized to weight the similarity matrix Z, aiming to describe the correlation between the similarity matrix and sequencing depth. This explicit consideration serves to minimize the influence of sequencing depth on the enhanced data. The specific formulation of this extended version of scCASE is as follows:

$$\min_{\mathbf{W,H,Z} \geq 0} F = \| \mathbf{X}(\mathbf{Z} \circ \mathbf{R}) - \mathbf{WH} \|_{\mathrm{F}}^2 + \lambda \| \mathbf{Z} - \mathbf{P}(\mathbf{H}^T\mathbf{H}) \|_{\mathrm{F}}^2 + \gamma_1 \| \mathbf{W} \|_{\mathrm{F}}^2 + \gamma_2 \| \mathbf{H} \|_{\mathrm{F}}^2 \tag{23}$$

We can convert the loss function from a norm form to a trace form as Equation (24), making it easier to compute the gradient.

$$\begin{gathered} F = tr((\mathbf{Z} \circ \mathbf{R})^T\mathbf{X}^T\mathbf{X}(\mathbf{Z} \circ \mathbf{R}) - \mathbf{H}^T\mathbf{W}^T\mathbf{X}(\mathbf{Z} \circ \mathbf{R}) - (\mathbf{Z} \circ \mathbf{R})^T\mathbf{X}^T\mathbf{WH} + \mathbf{H}^T\mathbf{W}^T\mathbf{WH}) \\ +\lambda tr(\mathbf{Z}^T\mathbf{Z} - \mathbf{P}(\mathbf{H}^T\mathbf{H})\mathbf{Z} - \mathbf{Z}^T(\mathbf{P}(\mathbf{H}^T\mathbf{H})) + (\mathbf{P}(\mathbf{H}^T\mathbf{H}))(\mathbf{P}(\mathbf{H}^T\mathbf{H}))) \\ +\gamma_1 tr(\mathbf{W}^T\mathbf{W}) + \gamma_2 tr(\mathbf{H}^T\mathbf{H}) \end{gathered} \tag{24}$$

After obtaining the partial derivatives of F with respect to $\mathbf{W}$, $\mathbf{H}$, and $\mathbf{Z}$ (Equation (25)), we use gradient descent to optimize the model. We initialize the step size to 0.2. If this

step size cannot reduce the loss function during the iteration process, we will reduce the step size and try updating them again.

$$\frac{\partial F}{\partial \mathbf{W}} = -2\mathbf{X}(\mathbf{Z} \circ \mathbf{R})\mathbf{H}^{\mathrm{T}} + 2\mathbf{W}\mathbf{H}\mathbf{H}^{T} + 2\gamma_1\mathbf{W}$$
$$\frac{\partial F}{\partial \mathbf{H}} = -2\mathbf{W}^{T}\mathbf{X}(\mathbf{Z} \circ \mathbf{R}) + 2\mathbf{W}^{T}\mathbf{W}\mathbf{H} - 2\lambda\mathbf{H}(\mathbf{Z}\mathbf{P} + (\mathbf{Z}\mathbf{P})^{T}) + 2\lambda\mathbf{H}(\mathbf{H}^{T}\mathbf{H}\mathbf{P}^{T}\mathbf{P} + \mathbf{P}^{T}\mathbf{P}\mathbf{H}^{T}\mathbf{H}) + 2\gamma_2\mathbf{H} \quad (25)$$
$$\frac{\partial F}{\partial \mathbf{Z}} = 2(\mathbf{X}^{T}\mathbf{X}(\mathbf{Z} \circ \mathbf{R})) \circ \mathbf{R} - 2(\mathbf{X}^{T}\mathbf{W}\mathbf{H}) \circ \mathbf{R} + 2\lambda\mathbf{Z} - 2\lambda\mathbf{P}\mathbf{H}^{T}\mathbf{H}$$

We validated the efficacy of this model in effectively correcting for sequencing depth. Using the same evaluation metrics, we applied SVD to the enhanced data and calculated the correlation coefficients between each component and sequencing depth. In the data enhanced by the extended scCASE, it is hard to observe a significant correlation between individual principal components and the sequencing depth (Supplementary Figs. S7c, f, S8c, f). Additionally, we conducted a clustering performance assessment for this enhanced method. The results suggest that the extension achieved enhancement performance similar to the original version, while successfully mitigating the impact of sequencing depth. We have made this model available as an optional variant of scCASE.

Supplementary Text S4: Statement and evaluation of cell type-specific peaks identified by scCASE.

Firstly, our description of downstream analysis aims to illustrate that scCASE is an interpretable method with the advantage of extracting biological insights for specific cell populations while enhancing scATAC-seq data. This is a unique feature that many other scCAS data enhancement methods cannot accomplish. For scCASE, we can consider a column of the projection matrix (a pattern of peaks) corresponding to the row of cell embedding with the highest activation levels in a certain cluster, investigate the pattern of peaks with relatively large coefficients, and identify the cell type-specific peaks. This illustrates that the scCASE model can learn peak accessibility patterns of cell types, providing more biological insights than other methods and demonstrating better interpretability.

Secondly, we comprehensively compared the cell type-specific peaks identified by scCASE and the differentially accessible peaks (DAPs) identified by EpiScanpy. Taking the Blood dataset as an example again, we computed the overlap between the scCASE-identified cell type-specific top 1000 peaks and the top 1000 DAPs identified by EpiScanpy. Supplementary Figure S15 illustrates a moderate degree of overlap between the cell type-specific peaks identified by scCASE and the DAPs identified by EpiScanpy. In six cell types, including CLP, CMP, LMPP, MEP, mono, and pDC, 50%-80% of the peaks are overlapped, and there is less degree of overlap in the cell types of HSC and GMP, respectively with 341 and 343 overlapping peaks.

Due to the limited overlap between cell type-specific peaks identified by scCASE and the

DAPs identified by EpiScanpy of HSC and GMP cells, we used these two types as an example to demonstrate how the specific peaks identified by scCASE contribute to superior biological insights into cellular heterogeneity. The two methods had four sets of peaks across the two cell types, including HSC-specific peaks identified by scCASE, GMP-specific peaks identified by scCASE, DAPs of HSC identified by EpiScanpy and DAPs of GMP identified by EpiScanpy. To investigate the differences in the peaks obtained by the two methods, we removed their intersection in each type, resulting in an additional four sets of peaks including scCASE-unique HSC-specific peaks, scCASE-unique GMP-specific peaks, the unique DAPs of HSC identified by EpiScanpy and the unique DAPs of GMP identified by EpiScanpy. Then we performed single-nucleotide polymorphisms (SNPs) enrichment analysis using SNPsea to obtain tissues explicitly affected by these peaks. Note that hematopoietic stem cells (HSCs) serve as the foundational source for immune cells, including T cells and B cells and GMP (granulocyte-macrophage progenitor) cells represent a stage in hematopoiesis and give rise to various immune cells[7-9]. HSCs and GMPs play a central role in orchestrating the generation and continuous replenishment of various immune cell types, contributing to the overall functionality of the immune system. Therefore, the HSC and GMP specificity peaks should exhibit a higher correlation with whole blood, myeloid cells, and lymphocytes. In the specific peaks obtained by scCASE, we can significantly observe this correlation (Supplementary Fig. S16a-d). However, the specific peaks unearthed by EpiScanpy do not capture this correlation effectively (Supplementary Fig. S16e-h). The genomic region enrichment of annotation tool (GREAT) analysis of scCASE-unique HSC-specific peaks obtained 20 pathways, comprehensively associated with functions such as immune regulation, immune cell activation,

hematopoietic regulation, etc. In contrast, the unique DAPs of HSC identified by EpiScanpy consist of only four pathways, solely related to immune regulation.

Finally, we applied EpiScanpy to the data enhanced by baseline methods to identify DAPs, and to compare the potential of scCASE and other baseline methods in uncovering biological insights. We first generated an upset plot for each group of peaks, and the results showed a higher intersection between EpiScanpy + raw data and scCASE, while the intersections between EpiScanpy + data enhanced by other methods were less prominent (Supplementary Fig. S17). To validate whether EpiScanpy can identify DAPs with greater biological specificity from data enhanced by baseline methods, we utilized DAPs identified by EpiScanpy on the raw data, DAPs identified by EpiScanpy on the data enhanced by scOpen, and cell type-specific peaks identified by scCASE as examples, given DAPs identified by EpiScanpy + scOpen had minimal overlap with EpiScanpy + raw data (Supplementary Fig. S17). We generated heatmaps using the raw data and different sets of peaks (Supplementary Fig. S18). Supplementary Figure S18a displays DAPs identified by EpiScanpy + raw data, the Supplementary Fig. S18b showcases DAPs identified by EpiScanpy + scOpen, and the Supplementary Fig. S18c presents cell type-specific peaks identified by scCASE. It was observed in Fig. R14b that when EpiScanpy was applied to the data enhanced by scOpen, although the specific peaks obtained were indeed specific, compared to the original data, these peaks exhibited low accessibilities in each type and higher randomness. Therefore, they are not the biological cell type-specific peaks we aimed to identify. In other words, the accessibilities of the identified peaks are limited, and they are only sporadically accessible in certain cells, rather than being specific to that particular cell type, holding less biological significance. The enhancement by scOpen

magnified such signals, which is not desired. Downstream analysis also confirmed that these peaks do not effectively reflect cellular heterogeneity. We implemented downstream analysis using cell type-specific peaks identified by scCASE and the DAPs identified by EpiScanpy + data enhanced by baseline methods (Supplementary Figs. S19, S20). With regard to SNPs enrichment analysis, HSC-specific peaks and GMP-specific peaks identified by scCASE demonstrate better cell type-specificity compared to EpiScanpy + data enhanced by baseline methods (Supplementary Fig. S19). The heritability enrichment analysis showed that the blood-related phenotypes exhibited higher associations with the HSC-specific peaks and GMP-specific peaks identified by scCASE than that of EpiScanpy + data enhanced by baseline methods (Supplementary Fig. S20).

Overall, the enrichment results with the cell type-specific peaks identified by scCASE are relatively better than those identified by EpiScanpy in the raw data or data enhanced by baseline methods, demonstrating the biological significance of the cell type-specific peaks identified by scCASE.

Supplementary Text S5: Details of extended scCASE model in enhancing data with batch effects.

We further investigated the potential of scCASE for removing batch effects. Assuming there is a total of n cells, we define B_j as the batch label of cell j and b_j as the number of cells which has the same batch label as cell j. In the similarity matrix $\mathbf{Z} \in \mathbb{R}^{n\times n}$, given a certain cell j, the mean similarity between cells within the same batch equals $(\sum_{B_i=B_j}^{n} \mathbf{Z}_{ij})/b_j$ and the mean similarity between cells within different batches equals to $(\sum_{B_i\neq B_j}^{n} \mathbf{Z}_{ij})/(n-b_j)$. Typically, for a certain cell, the mean similarity between cells within the same batch is significantly higher than that between different batches. To utilize cross-batch information for enhancement more effectively, we have introduced a fixed similarity matrix $\mathbf{Z}^{\mathrm{fix}} \in \mathbb{R}^{n\times n}$.

$$
\mathbf{Z}_{\mathrm{ij}}^{\mathrm{fix}} = \begin{cases} \mathbf{Z}_{\mathrm{ij}} \times \dfrac{\dfrac{\left(\sum_{B_k\neq B_j}^{n} \mathbf{Z}_{kj}\right)}{(n-b_j)}}{\dfrac{\left(\sum_{B_k=B_j}^{n} \mathbf{Z}_{kj}\right)}{b_j} + \dfrac{\left(\sum_{B_k\neq B_j}^{n} \mathbf{Z}_{kj}\right)}{(n-b_j)}}, \text{if } B_i = B_j, \\ \mathbf{Z}_{\mathrm{ij}} \times \dfrac{\dfrac{\left(\sum_{B_k=B_j}^{n} \mathbf{Z}_{kj}\right)}{b_j}}{\dfrac{\left(\sum_{B_k=B_j}^{n} \mathbf{Z}_{kj}\right)}{b_j} + \dfrac{\left(\sum_{B_k\neq B_j}^{n} \mathbf{Z}_{kj}\right)}{(n-b_j)}}, \text{if } B_i \neq B_j. \end{cases} \quad (26)
$$

In the extended scCASE, after the iterations of similarity matrix $\mathbf{Z}$, we weight it using Equation (26), replace $\mathbf{Z}$ with $\mathbf{Z}^{\mathrm{fix}}$, ensuring that the mean similarity of each cell with cells from the same batch equals that with cells from different batches and achieved a better enhancement of data with batch effects. We provide the extended version of scCASE as an optional variant.

Supplementary Text S6: Discussion of different peak filtering strategies.

In scCASE, the filtering of peaks and the threshold for filtering are treated as optional parameters. In other words, this is not a mandatory step. However, in the analysis of scCAS data, the standard workflow of EpiScanpy and the methods specifically designed for scCAS data usually include peak filtering during the preprocessing steps[4, 10-14]. This is because scCAS data often contains numerous peaks only accessible in very few cells, or even completely inaccessible in all cells. To validate the impact of peak filtering and different filtering thresholds on clustering outcomes, we conducted the following experiments. Firstly, we executed the scCASE method without peak filtering on eight datasets and evaluated both computational efficiency and clustering performance. The experimental results demonstrated that the clustering results using the unfiltered scCASE method and the scCASE method filtered with a default 1% threshold were relatively close. We performed a two-sided Wilcoxon signed-rank test on the clustering metrics of scCASE with a 1% filtering threshold and scCASE without filtering. The p-values for ARI were 0.84, and for AMI it was 0.64, which indicated that the 1% filtering threshold does not significantly impact clustering performance. Simultaneously, the removal of these peaks effectively accelerated the run-time and saved memory usage. Across the BM0828, Blood, and LungA datasets, a 1% filtering threshold results in an average reduction of 56% in run-time and 69% in peak memory usage.

Secondly, we also explored the impact of different filtering thresholds on scCASE. We ran scCASE with filtering thresholds set at 3% and 5% on the eight datasets, assessing both computational efficiency and clustering performance. The results indicated that compared to the 1% filtering threshold, using 3% and 5% thresholds significantly discarded valuable

information in the datasets, leading to a decrease in clustering metrics. We acknowledge that excessive filtering can indeed lead to information loss. However, it is crucial to emphasize that this is not a case of "the more, the better" or "the less, the better." All the experiments in our manuscript indicate that the default 1% filtering threshold is a reasonably suitable choice.

Finally, we assessed the impact of the filtering on the identification of cell type-specific peaks. We ran the scCASE method without peak filtering on the Blood dataset and utilized scCASE to identify 1000 specific peaks for each cell type. Next, we examined whether those cell type-specific peaks were discarded when using a 1% filtering threshold. In most cases, the 1000 cell type-specific peaks obtained by the unfiltered scCASE method were not discarded during the filtering process; 99.56% of peaks were saved on average. This indicates that the filtering strategy does not significantly impact the identification of cell type-specific peaks.

In conclusion, firstly, the threshold for filtering is treated as an optional parameter in scCASE, allowing users to choose whether to perform filtering based on their specific needs. Secondly, filtering for the scCAS count matrix is a widely employed strategy, and our experiments demonstrate that a default 1% filtering threshold can decrease run-time and memory usage without affecting cell type identification and downstream analyses.

Supplementary Text S7: Discussion of differences and advantages of scCASE to PCA/SVD.

We will elaborate on the advantages of scCASE in scCAS data enhancement from three key aspects: 1) the algorithmic principles, characteristics, and advantages of NMF compared to PCA and SVD, 2) the distinctions between data enhancement methods such as scCASE and data dimensionality reduction methods such as PCA and SVD, and 3) the innovative aspects of scCASE building upon NMF.

Firstly, the PCA process involves finding a set of standard orthogonal bases, where the first base represents the direction with the highest variance in the original dataset. Subsequently, each subsequent base is chosen to be orthogonal to the previous ones and captures the maximum variance in its direction. Therefore, each dimension in PCA only represents the magnitude of variance, lacking clear physical interpretation for each base. When applied to scCAS data, PCA can reduce the dimensionality of the data but fails to extract specific cell type-chromatin accessibility patterns. The elements in the matrix obtained through PCA can be positive or negative, and since chromatin accessibility states are binary (open or closed) and the negative values cannot effectively describe this, making it challenging to interpret the results of PCA. Similarly, PCA can be seen as a specific application of SVD, sharing similar characteristics. The principal components obtained through PCA are essentially the left singular vectors of the data matrix. Both PCA and SVD are effective for data dimensionality reduction, but they are less commonly applied to data enhancement tasks. The standard workflow in EpiScanpy also provides steps using PCA for dimensionality reduction rather than enhancement. We performed imputation on various datasets using PCA and SVD, followed the

evaluation workflow as scOpen, and conducted clustering based on the low-dimensional representations. We assessed the clustering metrics, and Supplementary Fig. S30 indicated that compared with scCASE, they also fall short of achieving effective data enhancement.

In contrast to PCA and SVD, NMF decomposes data into latent components and has clear interpretability as basis vectors for the latent factors. For example, in the scCASE model, the projection matrix (**W**) stores weights regarding the peak-component, while the cell embedding matrix (**H**) holds weights for the cell-component. In this context, the chromatin accessibility information for each cell is represented as a weighted combination of multiple components. Moreover, NMF breaks down a non-negative matrix into the product of lower-rank non-negative matrices, approximating the original matrix while preserving this non-negativity. It means that NMF optimizes the reconstruction loss, similar to autoencoders, which is distinct from PCA and LSI. Non-negativity constraint also promotes the imputation of missing signals, as signals are explained additively in one direction. If negative values are permitted in the models, the algorithm may be tempted to subtract away real data points rather than imputing missing points. These characteristics make NMF commonly utilized in imputation tasks[15-18].

Secondly, we would like to emphasize that scCASE is a scCAS data enhancement method designed primarily to address dropout events in scCAS data. Its purpose is to impute and denoise the original scCAS data, focusing on preserving the data's original dimensions rather than achieving dimensionality reduction. In contrast, PCA and SVD are employed for data dimensionality reduction, providing a low-dimensional representation of cells that can be utilized for downstream analyses such as cell clustering. These two types of methods are not mutually exclusive but can complement each other. For instance, scCASE acts as a

preprocessing step to enhance the original data in its original dimensions, while PCA can be subsequently applied to the enhanced data for dimensionality reduction, obtaining a cell embedding. In our manuscript, following the approach of scOpen, we utilized data enhancement methods to impute the original data and then applied PCA to the enhanced data to obtain the cell embedding, followed by clustering and evaluation of the cell embedding.

Finally, scCASE is not a simple NMF model. In the modeling process, scCASE additionally considers the similarity between cells and creates a cell similarity matrix to smooth the data using cell similarity. This means that the chromatin accessibility state of a cell can be represented as the weighted average of the accessibility states of similar cells. Through multiple iterations, the projection matrix, cell embedding matrix, and similarity matrix are continuously updated, combining this information to achieve data enhancement. Moreover, scCASER is capable of incorporating publicly available omics data as reference data, providing prior knowledge to better characterize the target scCAS data and facilitate data enhancement.

Supplementary Figures

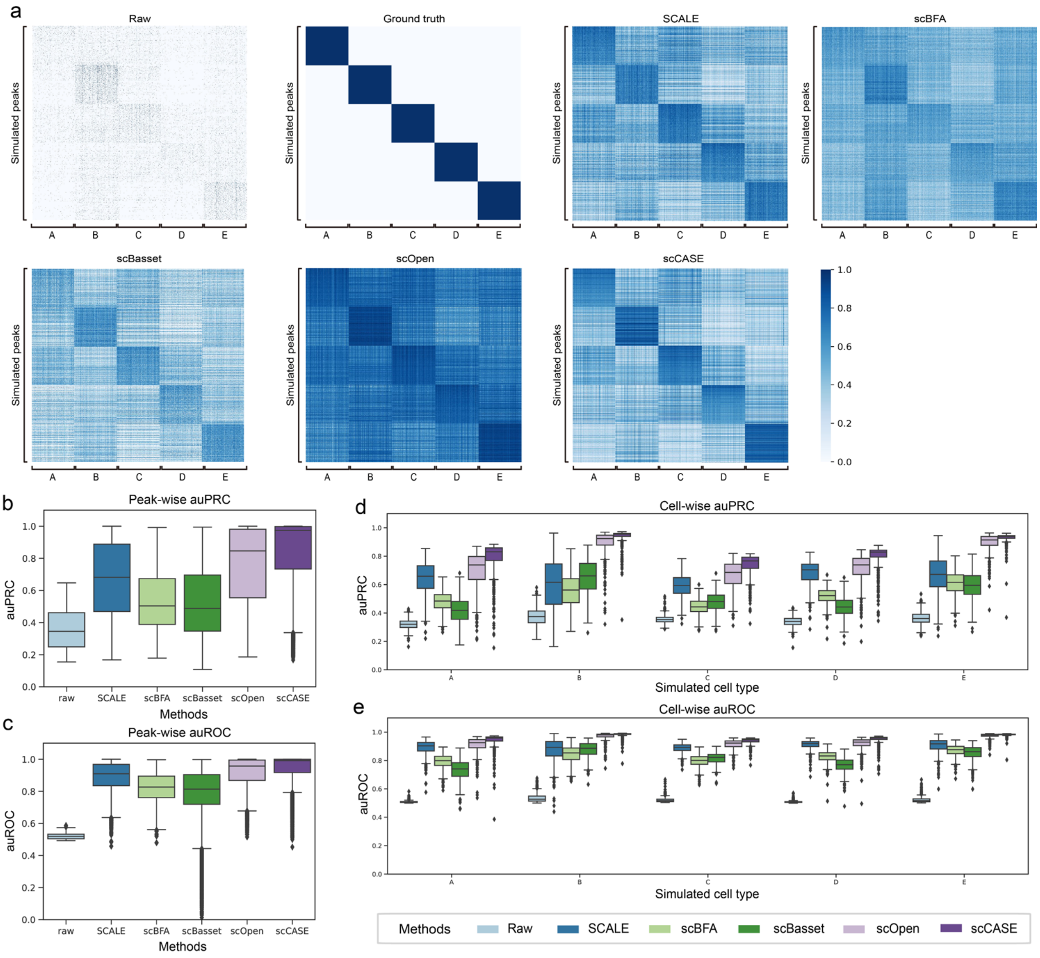

Supplementary Figure S1. The enhancement performance of scCASE and other scCAS enhancement methods. a, Heatmap of the simulated scCAS count matrix and the simulated data enhanced by various methods. The y-axis represents different peaks and the x-axis represents different cells. **b**, The boxplot of the peak-wise auPRC between the ground-truth simulated data and the data enhanced by different methods (n=15000 peaks). **c**, The boxplot of the peak-wise auROC between the ground-truth simulated data and the data enhanced by different methods (n=15000 peaks). **d**, The boxplot of the auPRC between the ground-truth simulated data and the data enhanced by different methods of each cell type (n=500 cells for each cell type). **e**, The boxplot of the auROC between the ground-truth simulated data and the data enhanced by different methods of each cell type. The midline represents the median, the boxes represent the interquartile range, whiskers represent 1.5× interquartile and points represent outliers (n=500 cells for each cell type). Source data are provided as a Source Data file.

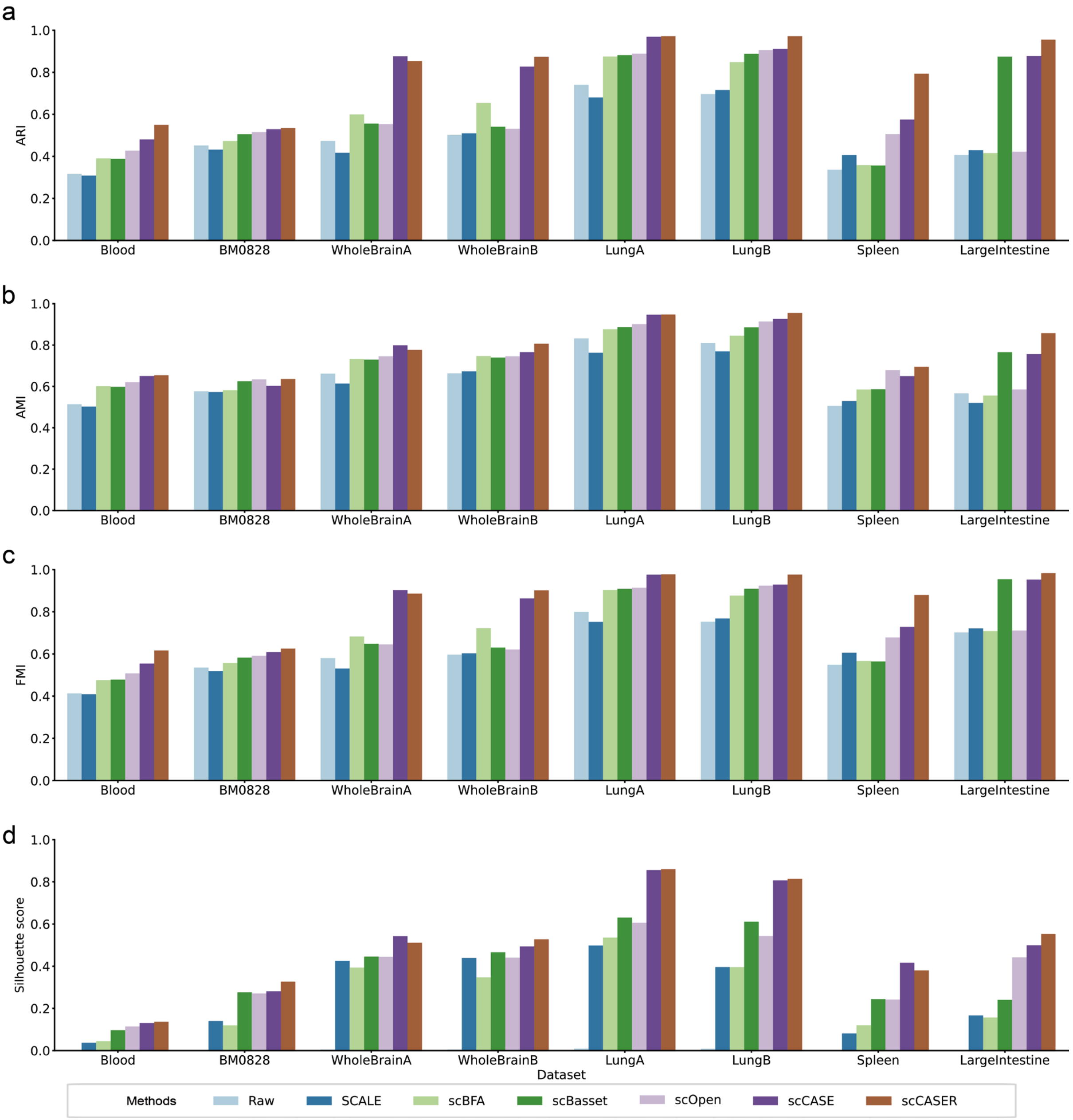

Supplementary Figure S2. A graphical illustration of the scCASE model and the benchmarking results of different methods. The clustering performance was assessed by **a**, ARI, **b**, AMI and **c**, FMI, scores on eight datasets enhanced by various methods. **d**, Silhouette scores according to cell type labels on eight datasets enhanced by various methods. Source data are provided as a Source Data file.

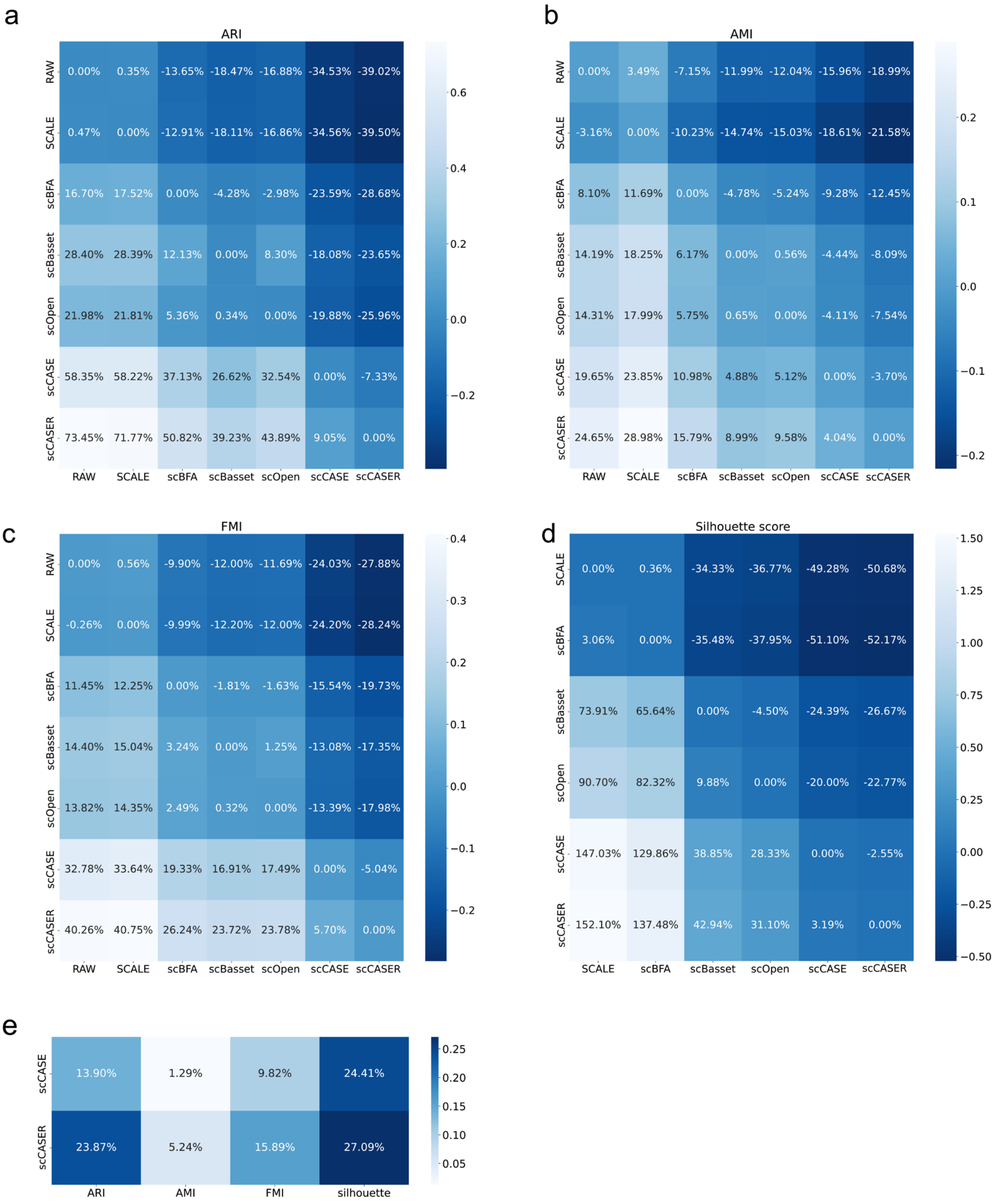

Supplementary Figure S3. Performance improvement of scCASE and other methods. a-d, The average improvement in values of different metrics between one method (one of the column names) and another (one of the row names) on eight datasets. Raw data is not shown in Supplementary Fig. S3d because its silhouette score is too low to be a denominator, avoiding extremely enormous values. **e**, The average improvement in clustering scores between scCASE/scCASER and the second-best method for each dataset.

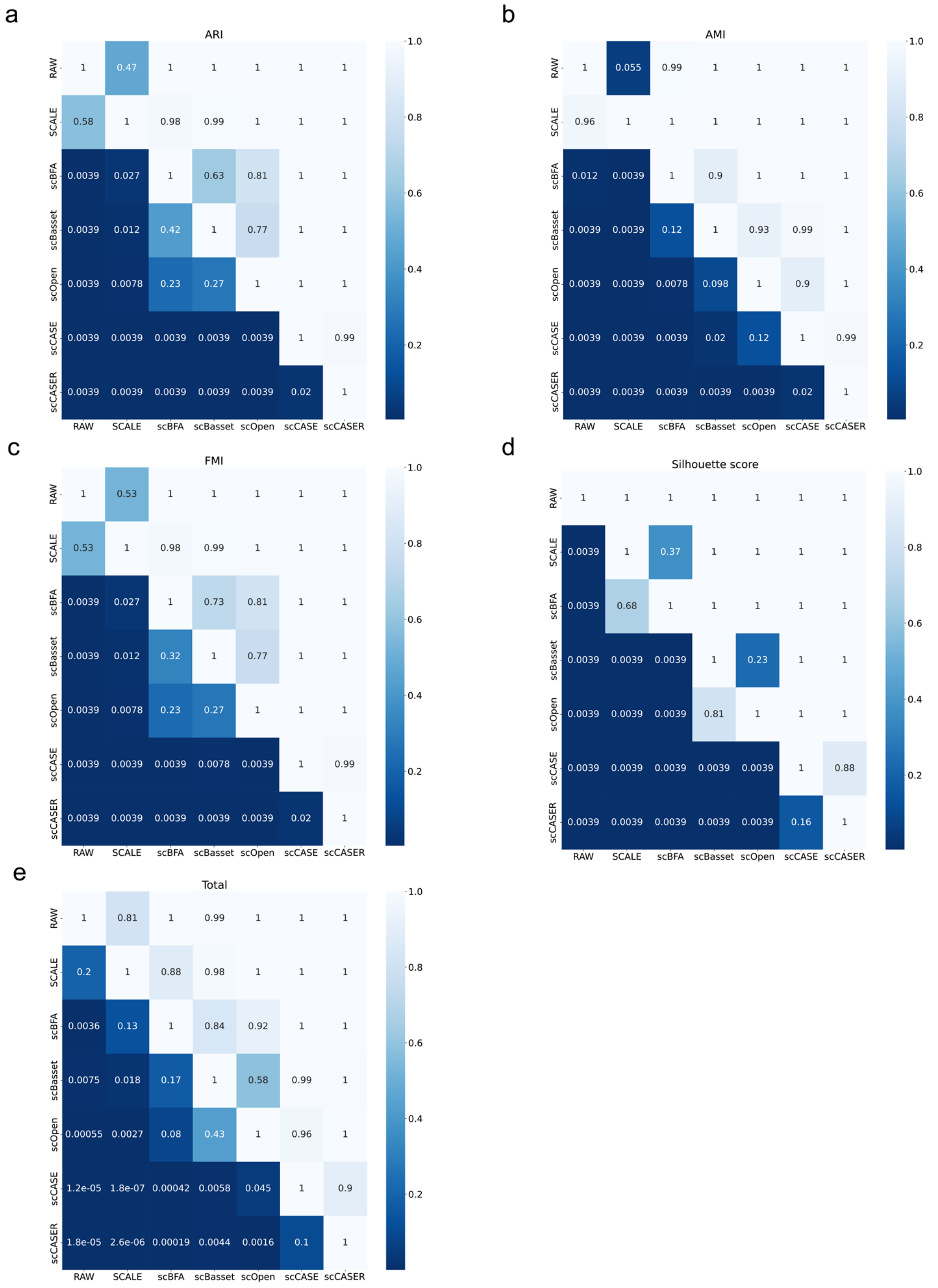

Supplementary Figure S4. Performance comparison of scCASE and other methods. a-d, *p*-values were obtained from one-sided paired Wilcoxon signed-rank tests to determine if one method (one of the column names) achieves significantly higher values of different metrics on the eight datasets (n=8) than another method (one of the row names). It is important to note that the lowest *p*-value of the test in eight samples is 0.0039. **e**, *p*-values of one-sided paired Wilcoxon signed-rank tests with eight datasets and four evaluating metrics (32 samples total).

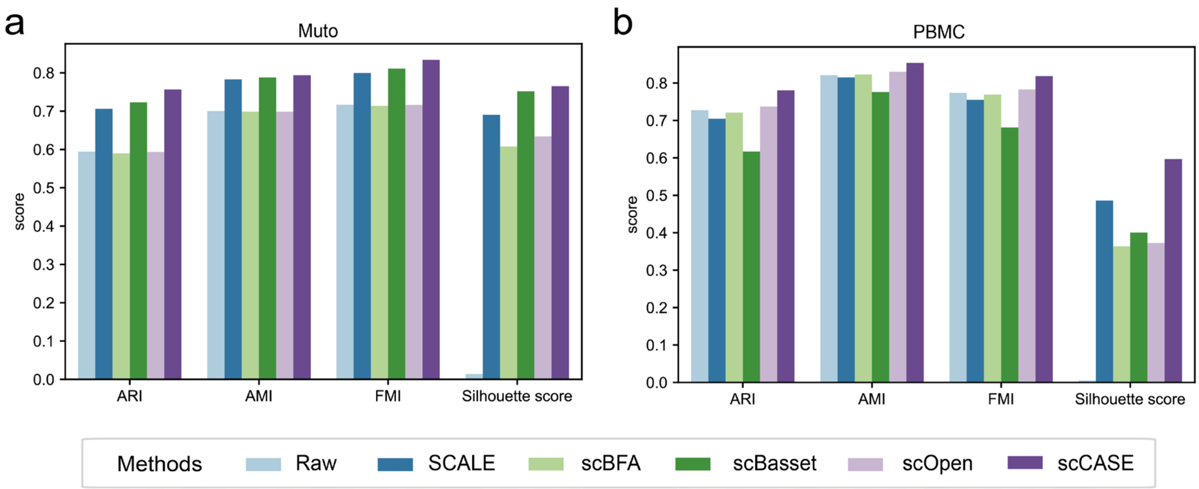

Supplementary Figure S5. Evaluation of clustering performance on the Muto and the PBMC datasets. a, the clustering values of different metrics of the Muto dataset. **b,** the clustering values of different metrics of the PBMC dataset. Source data are provided as a Source Data file.

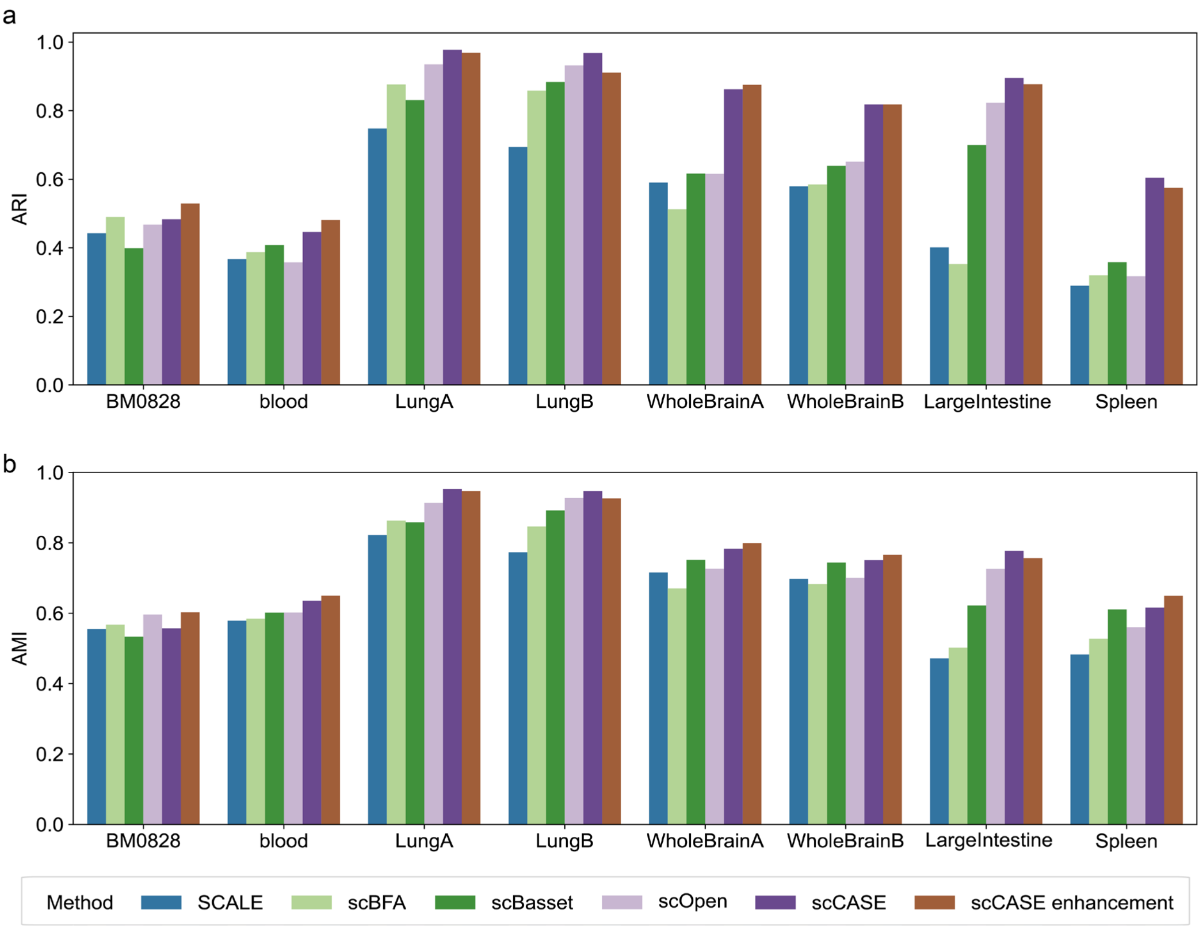

Supplementary Figure S6. Evaluation of clustering performance using cell embeddings of scCASE and other scCAS data enhancement methods. a, The clustering performance evaluated by ARI. **b,** The clustering performance evaluated by AMI. The term "scCASE enhancement" refers to clustering based on scCASE enhanced data + PCA. Source data are provided as a Source Data file.

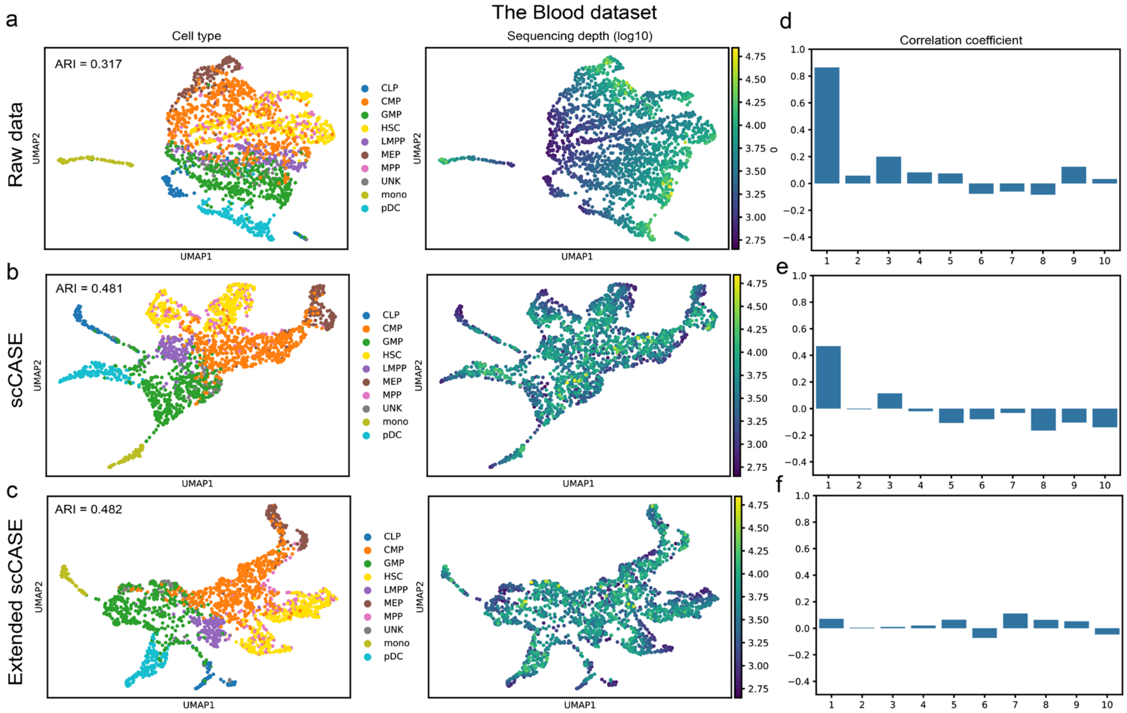

Supplementary Figure S7. Impact of sequencing depth on raw data and the data enhanced by scCASE on the Blood dataset. UMAP visualization of **a,** raw data, **b,** data enhanced by scCASE and **c,** data enhanced by the extended scCASE. **d-f,** Correlation coefficient between sequencing depth and SVD components of raw data, the data enhanced by scCASE and the extended scCASE, respectively, totally 2034 cells. Source data are provided as a Source Data file.

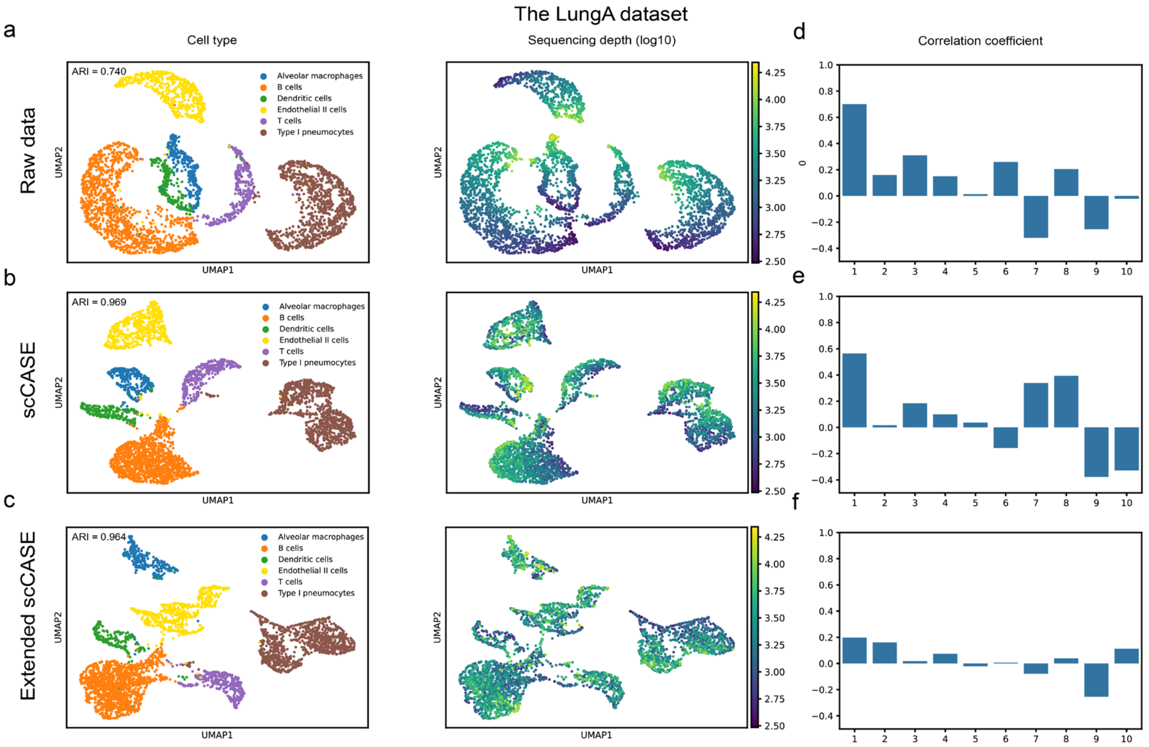

Supplementary Figure S8. Impact of sequencing depth on raw data and the data enhanced by scCASE on the LungA dataset. UMAP visualization of **a,** raw data, **b,** data enhanced by scCASE and **c,** data enhanced by the extended scCASE. **d-f,** Correlation coefficient between sequencing depth and SVD components of raw data, the data enhanced by scCASE and the extended scCASE, respectively, totally 3671 cells. Source data are provided as a Source Data file.

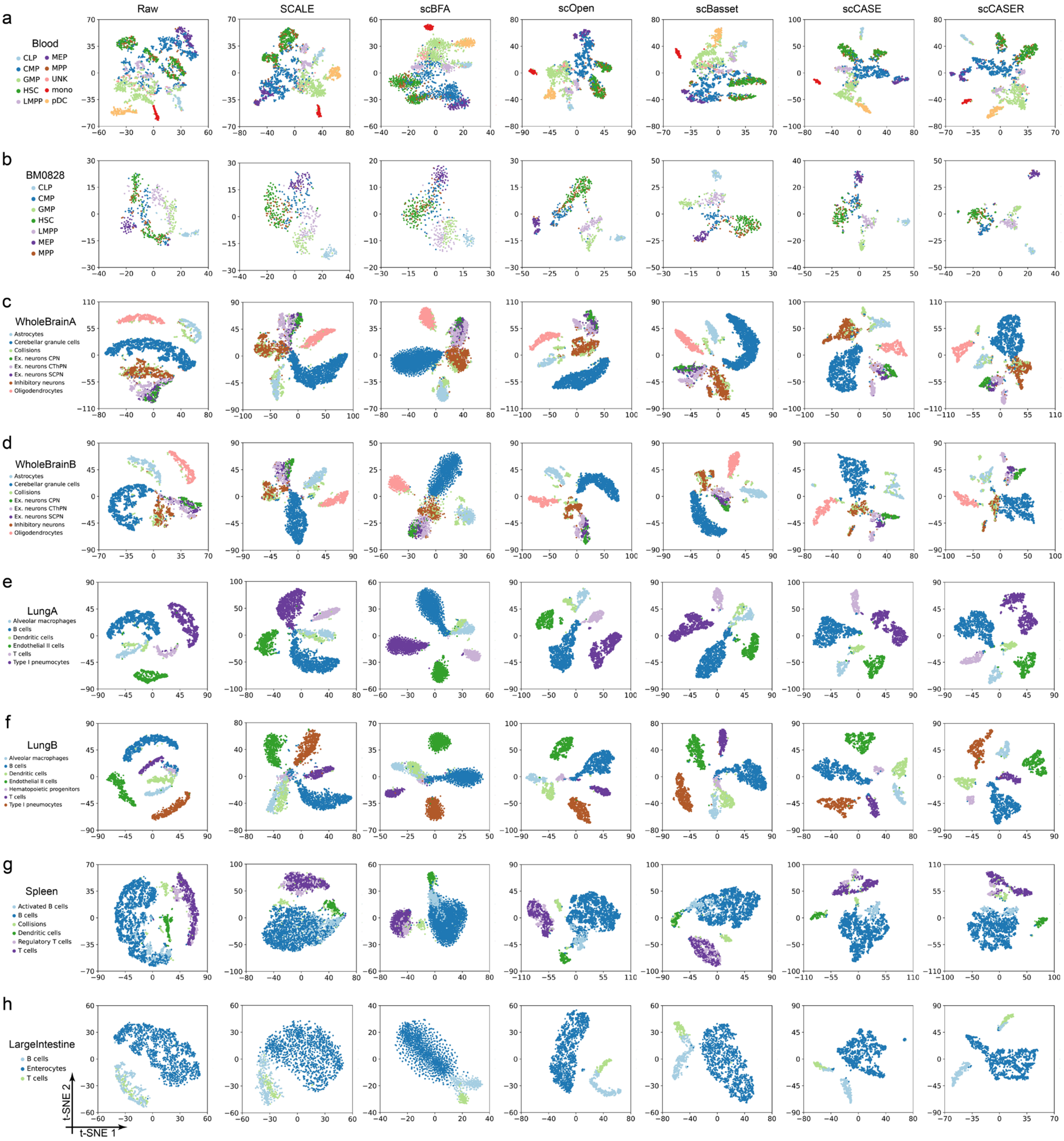

Supplementary Figure S9. t-SNE visualization of the raw scCAS data and the data enhanced by different methods. **a**, The Blood dataset. **b**, The dataset of BM0828. **c**, The WholeBrainA dataset. **d**, The WholeBrainB dataset. **e**, The LungA dataset. **f**, The LungB dataset. **g,** The Spleen dataset. **h**, The LargeIntestine dataset. Source data are provided as a Source Data file.

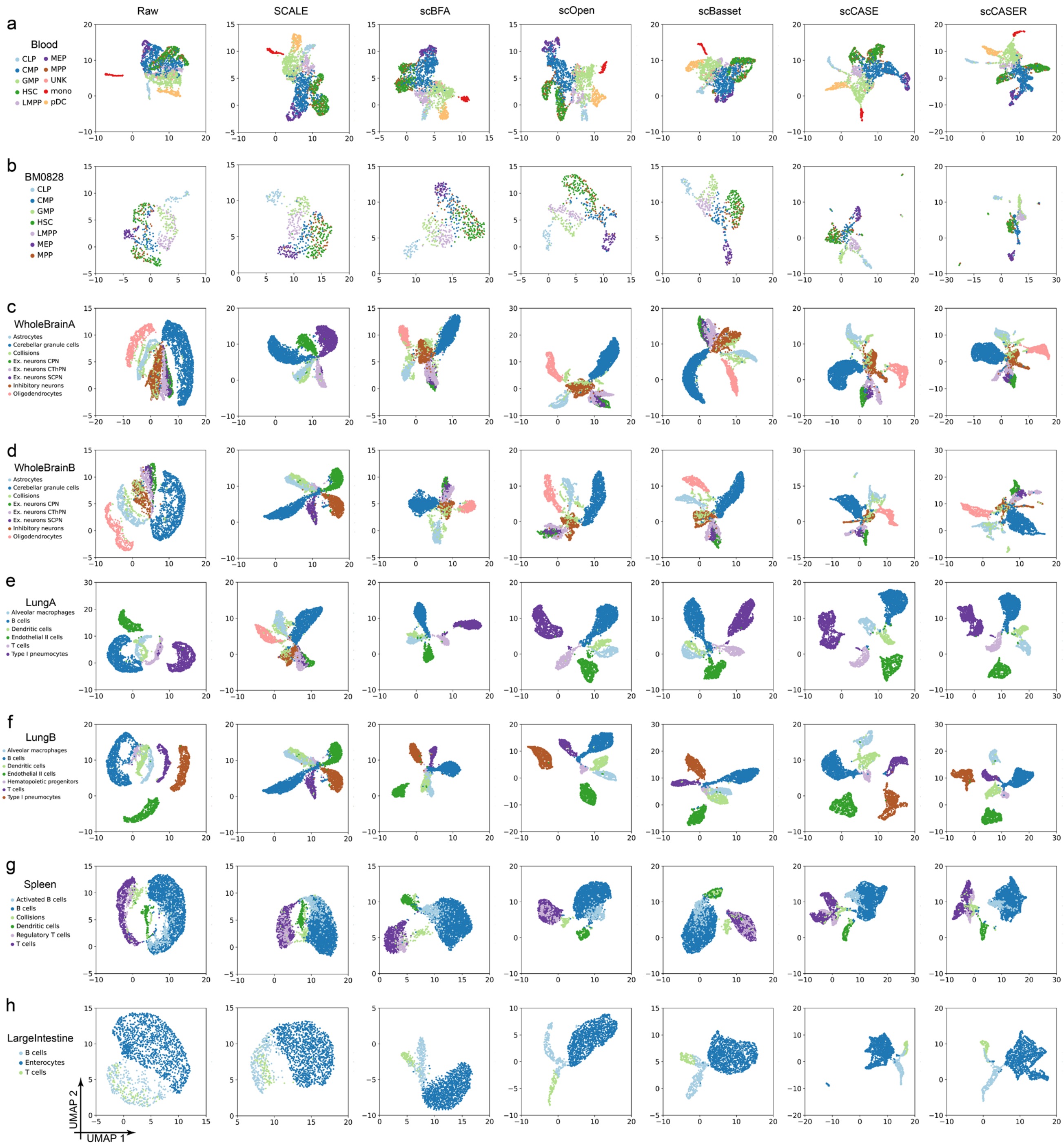

Supplementary Figure S10. UMAP visualization of the raw scCAS data and the data enhanced by different methods. **a**, The Blood dataset. **b**, The dataset of BM0828. **c**, The WholeBrainA dataset. **d**, The WholeBrainB dataset. **e**, The LungA dataset. **f**, The LungB dataset. **g,** The Spleen dataset. **h**, The LargeIntestine dataset. Source data are provided as a Source Data file.

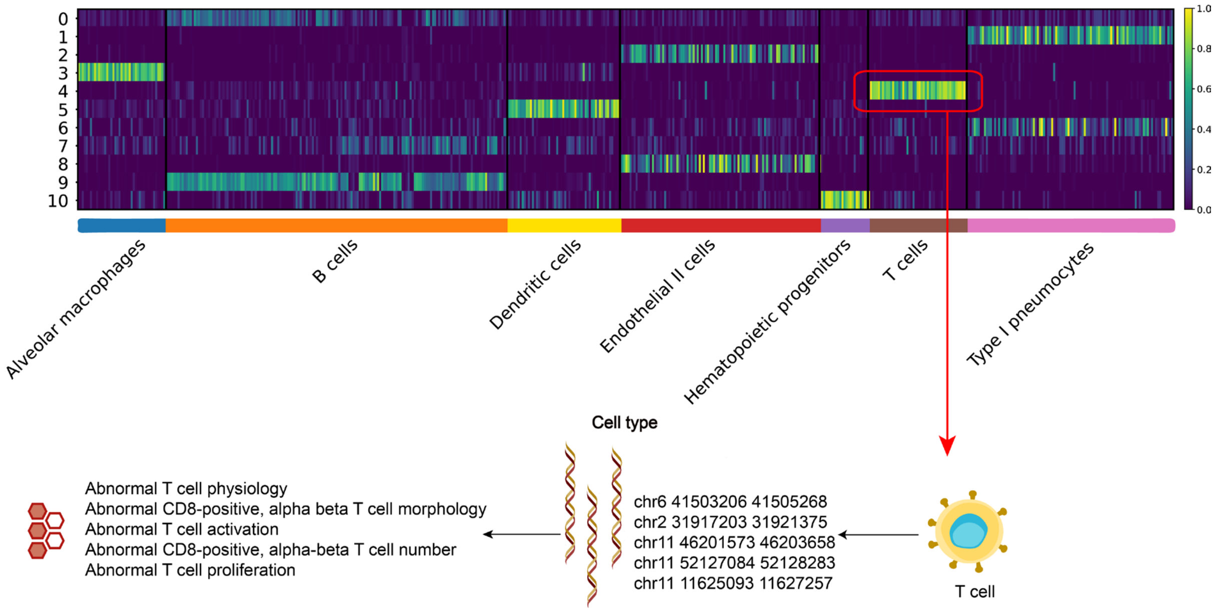

Supplementary Figure S11. The heatmap of the cell embeddings in latent space. We considered a column of the projection matrix that corresponds to the row of cell embedding with the highest activation levels in the monocyte cluster and identified the top 100 T-cell specific peaks as of the LungB dataset. The enriched pathways were consistent with their known functions. Source data are provided as a Source Data file.

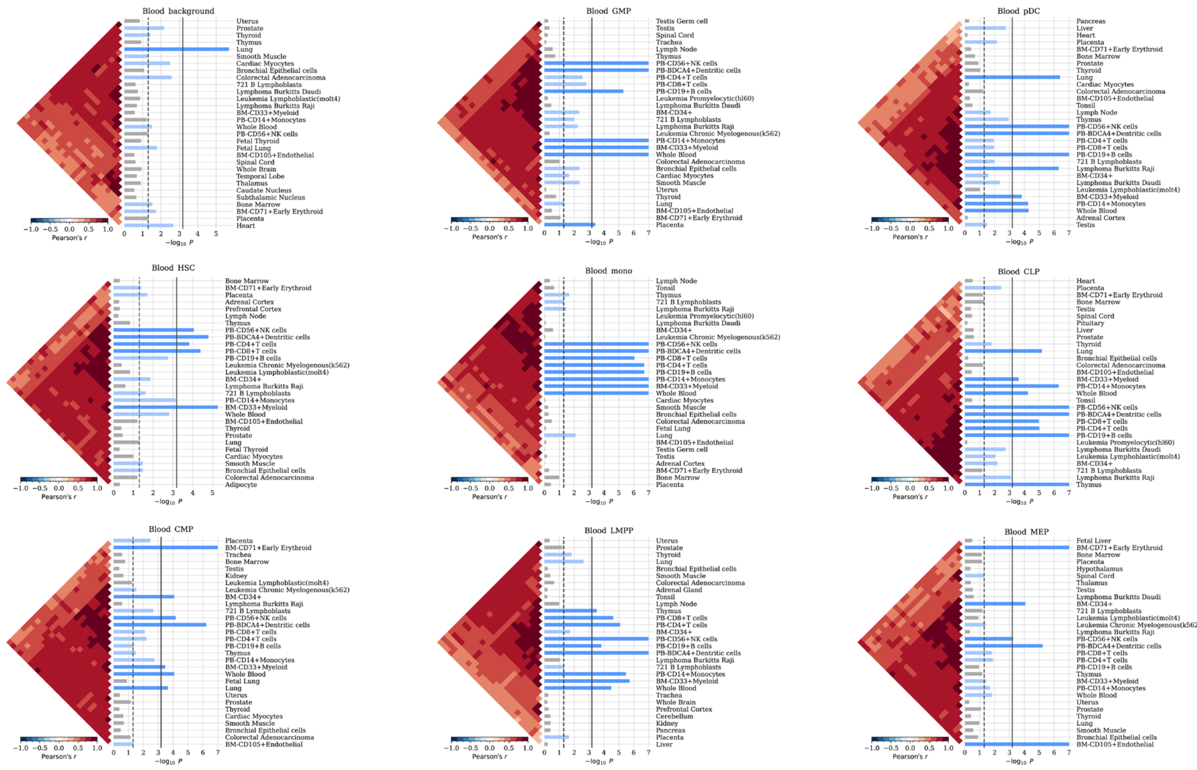

Supplementary Figure S12. Tissue-specific expression enrichment of the cell type-specifc peaks identified of scCASE and background peaks of the Blood dataset. Cell type specific peaks and background peaks were subjected to SNPsea analysis to determine the top 30 significantly enriched tissues. Bars represent the empirical p-values calculated by the SNPsea algorithm. The significance of gene enrichment for a specific annotation was assessed using one-sided *p*-value cutoffs at the 0.05 level, represented by vertical dashed and solid lines. The unadjusted *p*-value cutoff was used for the dashed line, while the Bonferroni-corrected *p*-value cutoff was applied for the solid line. The heatmaps display the Pearson correlation coefficients, which measure the similarity between expression profiles. The expression profiles were ordered using hierarchical clustering with the unweighted pair-group method with arithmetic means (UPGMA). Source data are provided as a Source Data file.

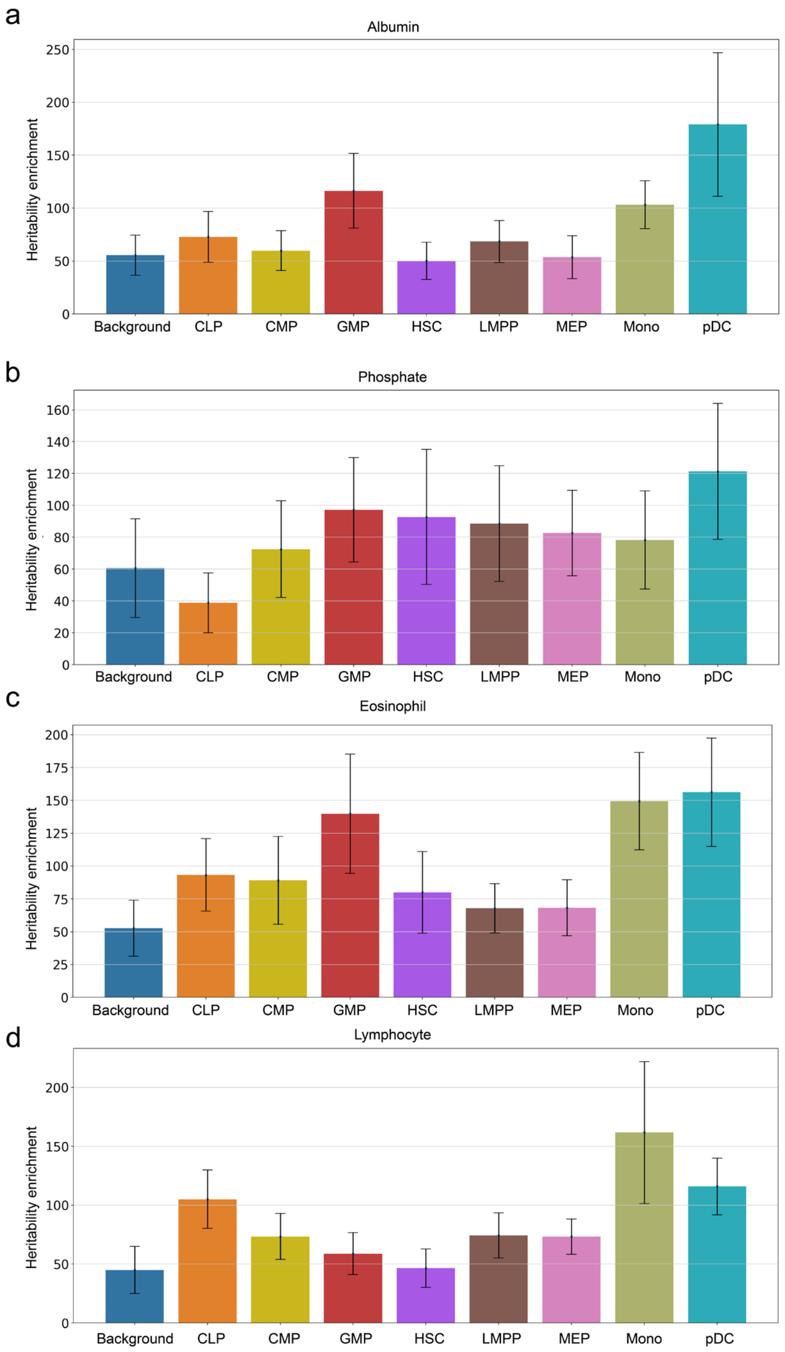

Supplementary Figure S13. Heritability enrichment of the cell type-specifc peaks identified of scCASE and background peaks of the Blood dataset. Stratified LDSC of the SNPs estimated heritability enrichment within the cell type-specific and background peaks for four blood-related traits. The error bars and centers of error bars represent the standard errors and average values of 200 equally sized jackknife blocks of adjacent SNPs about the estimates of enrichment. **a**, The phenotype of albumin. **b**, The phenotype of phosphate. **c**, The phenotype of eosinophil. **d**, The phenotype of lymphocyte. Source data are provided as a Source Data file.

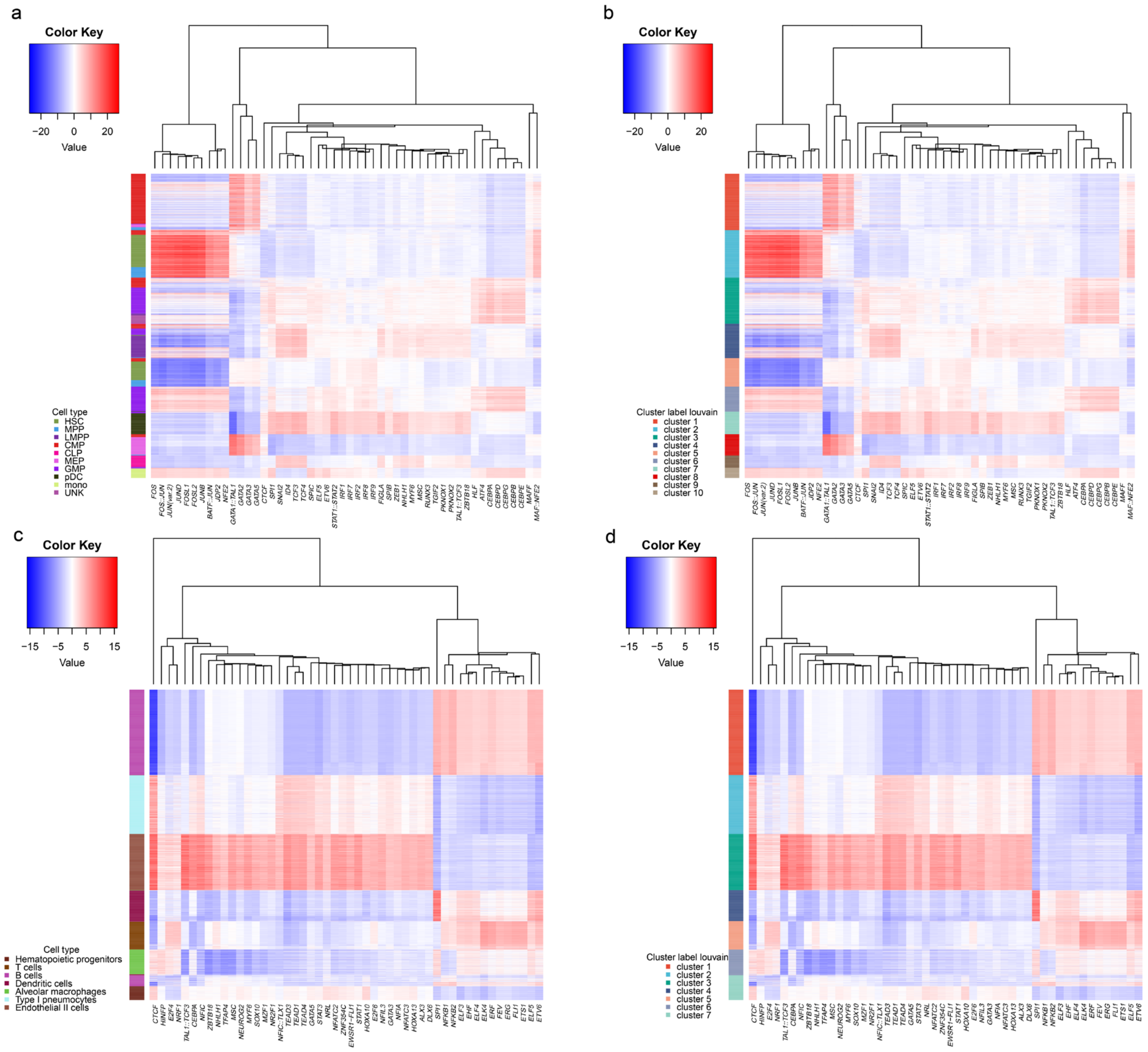

Supplementary Figure S14. Motif enrichment analysis of the scCAS data enhanced by scCASE. The top 50 most variable TF binding motifs within the cluster-specific peaks for the cells of the Blood and LungB datasets. The deviations calculated by chromVAR are shown. **a**, The Blood dataset with the labels of cell type. **b**, The Blood dataset with the labels of Louvain clustering results. **c**, The LungB dataset with the label of cell type. **d**, The LungB dataset with the label of Louvain cluster. Source data are provided as a Source Data file.

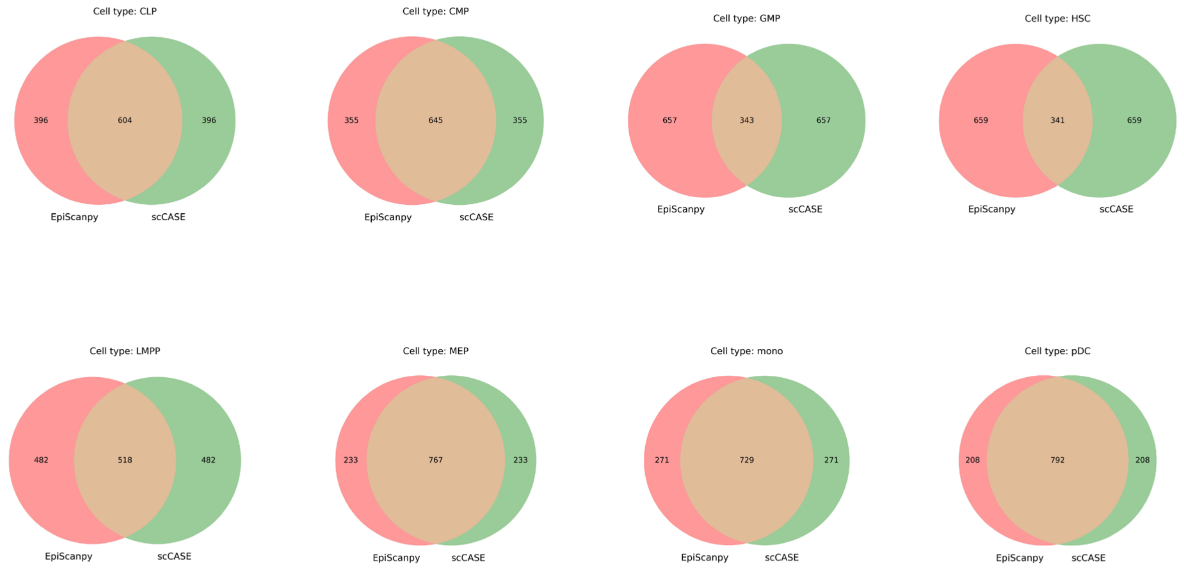

Supplementary Figure S15. The overlap of cell type-specific peaks identified by scCASE and the differentially accessible peaks identified by EpiScanpy on the raw data. Source data are provided as a Source Data file.

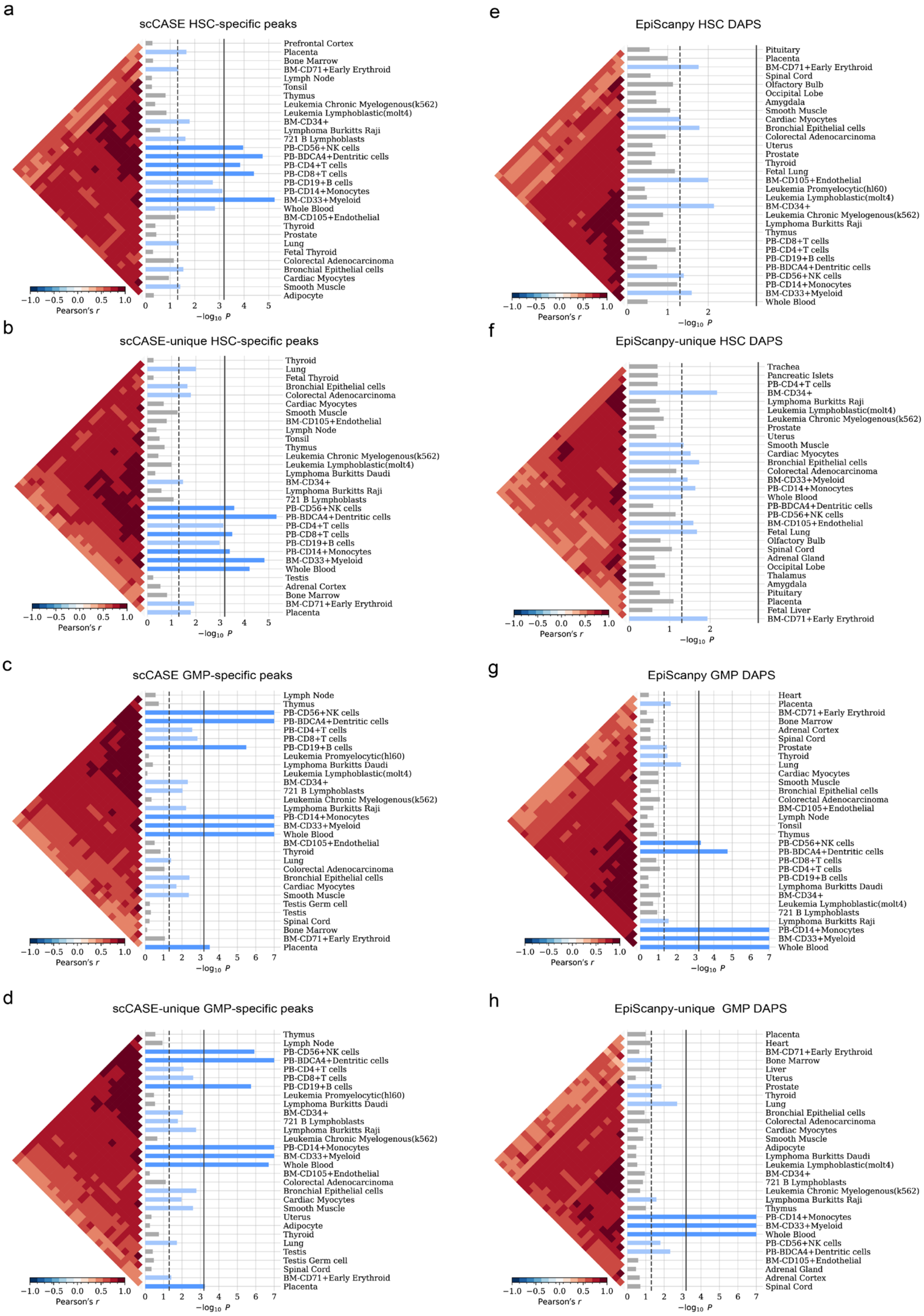

Supplementary Figure S16. SNPsea enrichment analysis for the cell type-specific peaks identified by scCASE and the differentially accessible peaks identified by EpiScanpy on the raw data. a-d, scCASE-identified cell type-specific peaks. **e-h,** DAPs identified by EpiScanpy. More explanations are provided in the legend of Supplementary Figure S12. Source data are provided as a Source Data file.

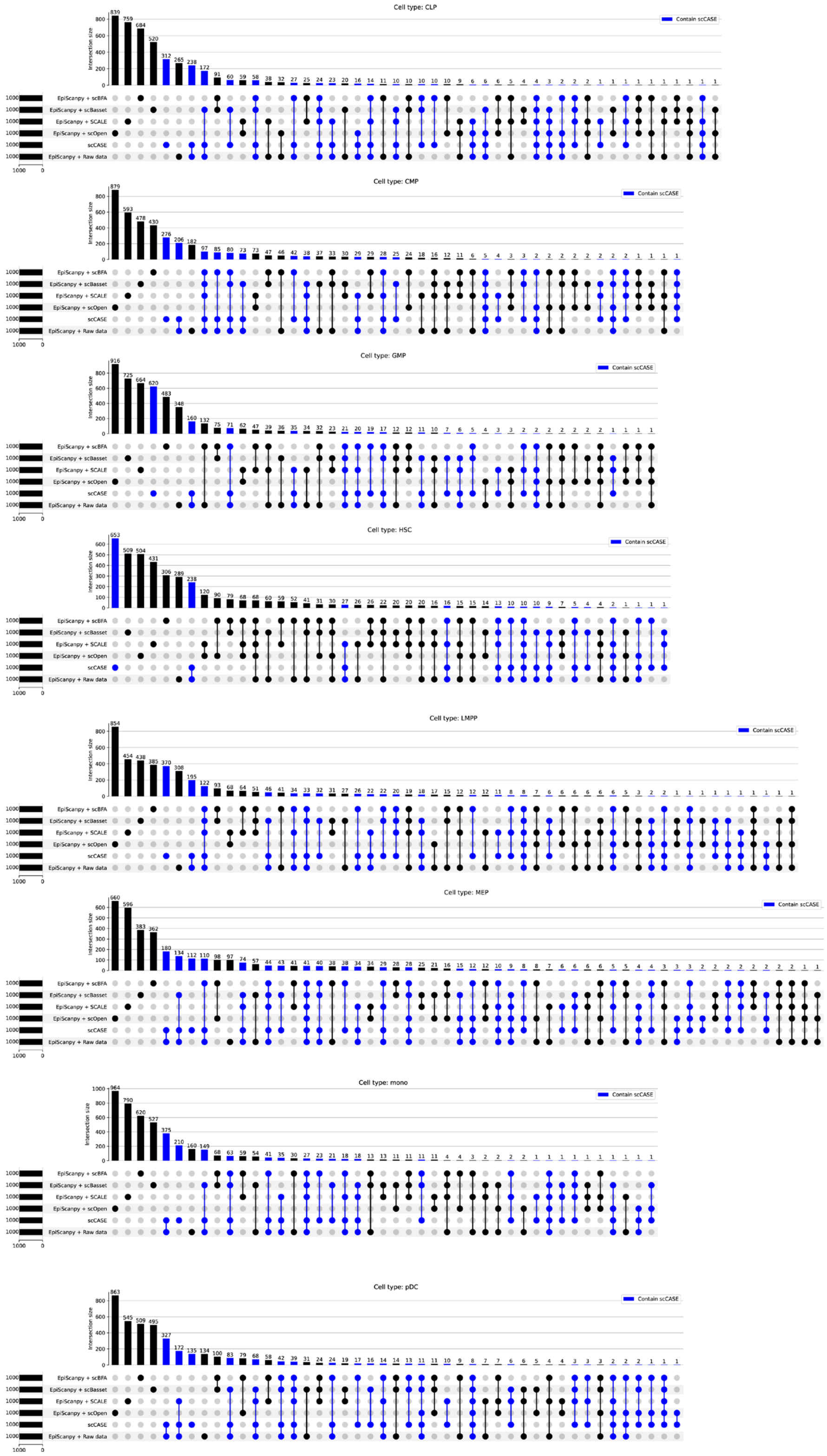

Supplementary Figure S17. The overlap of cell type-specific peaks identified by scCASE and the differentially accessible peaks identified by EpiScanpy on the raw data and the data enhanced by baseline methods. Source data are provided as a Source Data file.

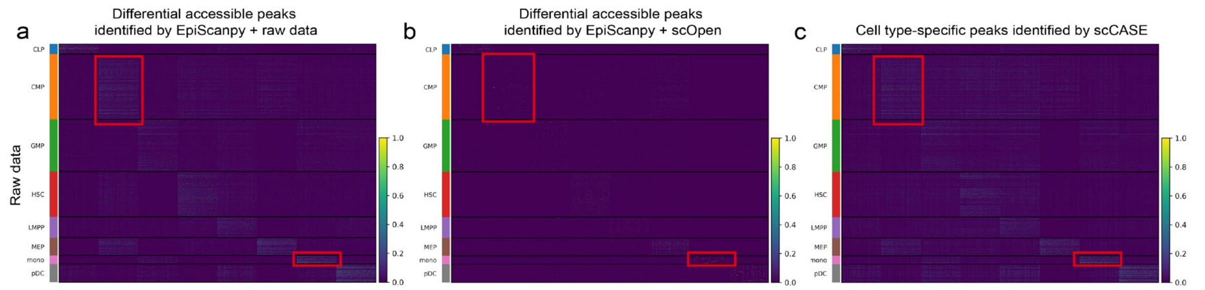

Supplementary Figure S18. Cell-peak heatmap of the raw data. a, Differential accessible peaks identified by EpiScanpy on the raw data. **b,** Differential accessible peaks identified by EpiScanpy on the data enhanced by scOpen. **c,** Cell type-specific peaks identified by scCASE. Source data are provided as a Source Data file.

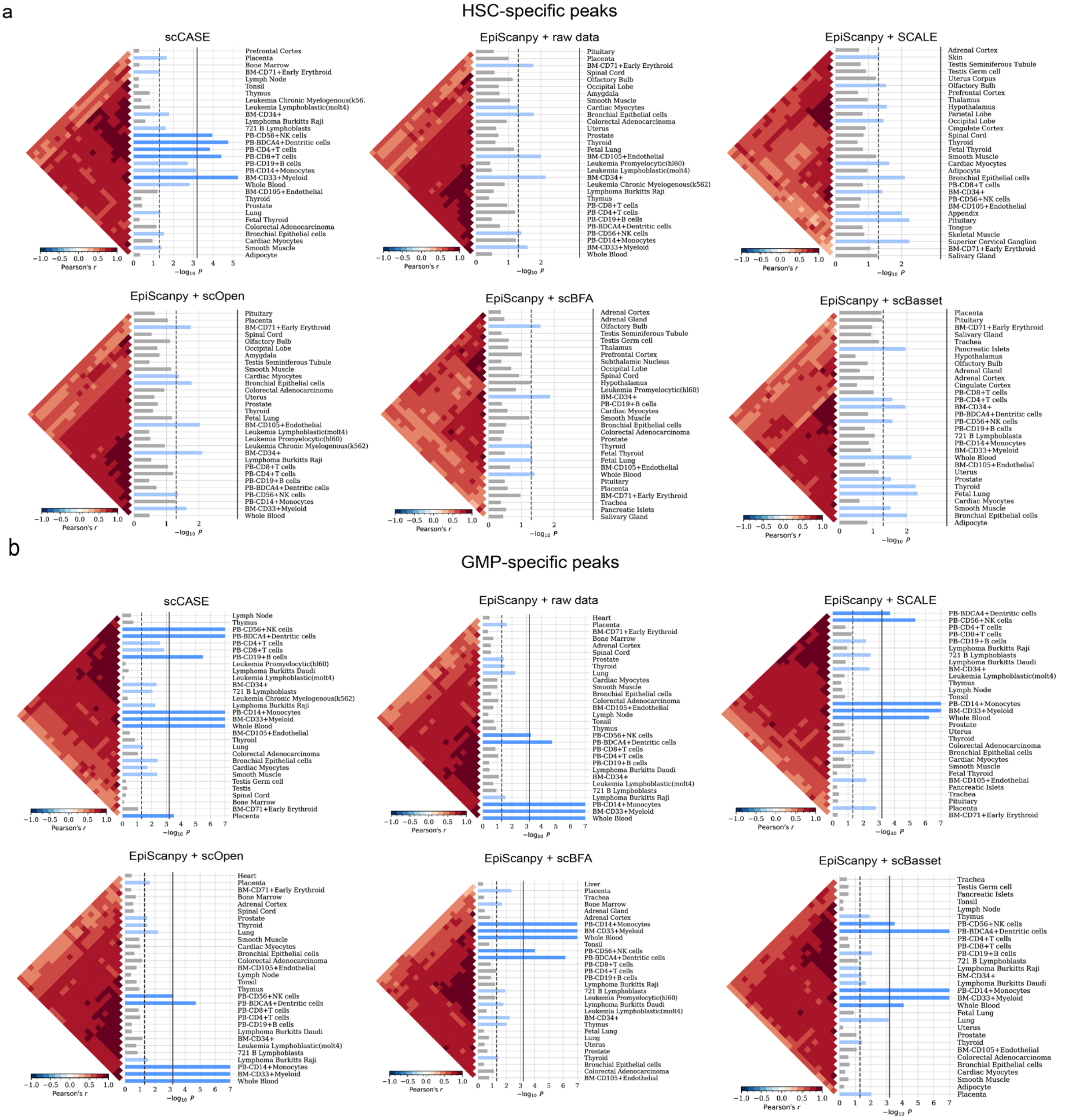

Supplementary Figure S19. SNPsea analysis for the scCASE-identified cell type-specific peaks and accessible peaks identified by EpiScanpy on raw data and the data enhanced by baseline methods. a, HSC-specific peaks. **b,** GMP-specific peaks. More explanations are provided in the legend of Supplementary Figure S12. Source data are provided as a Source Data file.

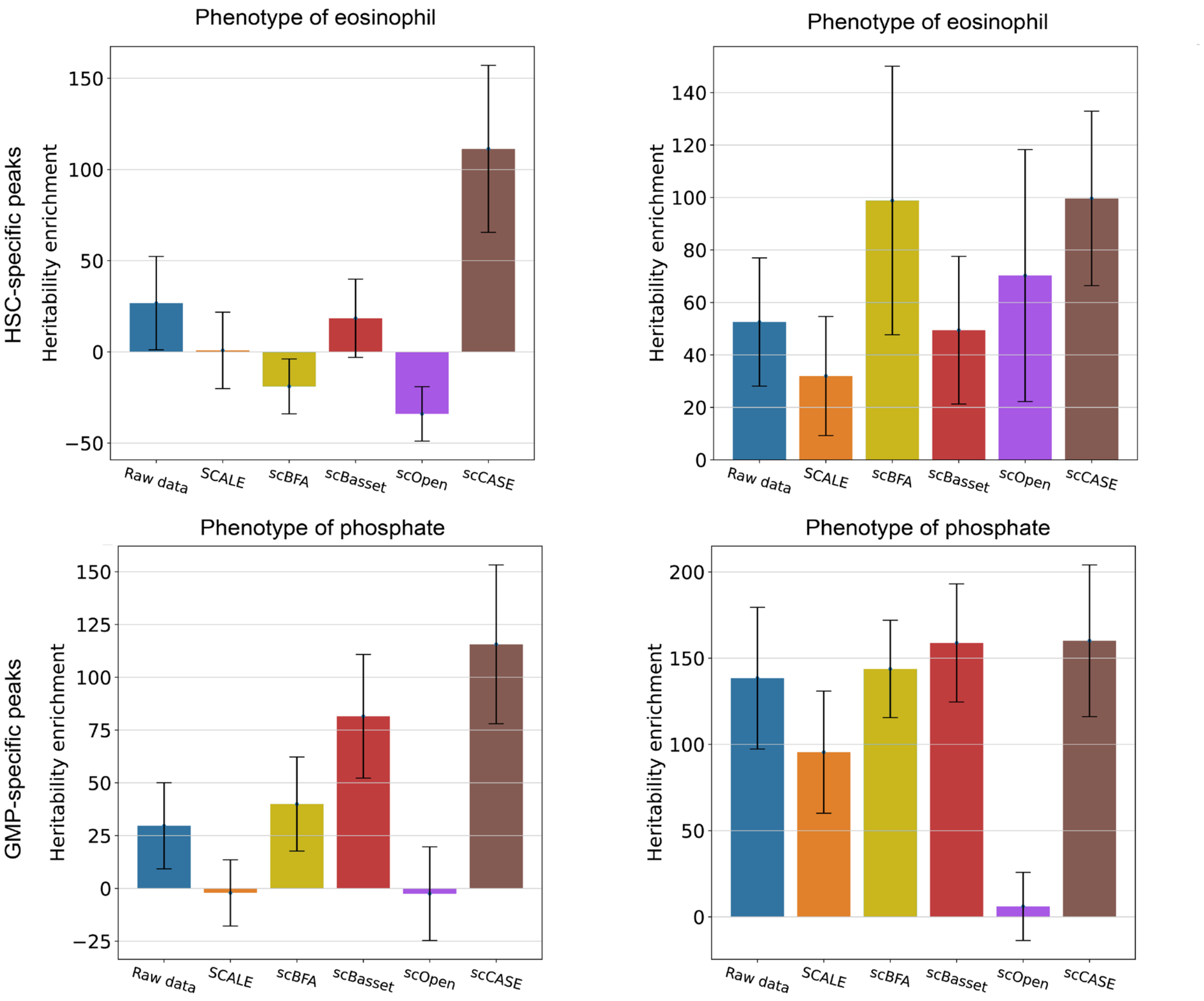

Supplementary Figure S20. Heritability enrichment estimates for the cell type-specific peaks identified by scCASE and accessible peaks identified by EpiScanpy on the raw data and the data enhanced by baseline methods. The error bars and centres of error bars represent the standard errors and average values of 200 equally sized jackknife blocks of adjacent SNPs about the estimates of enrichment. Source data are provided as a Source Data file.

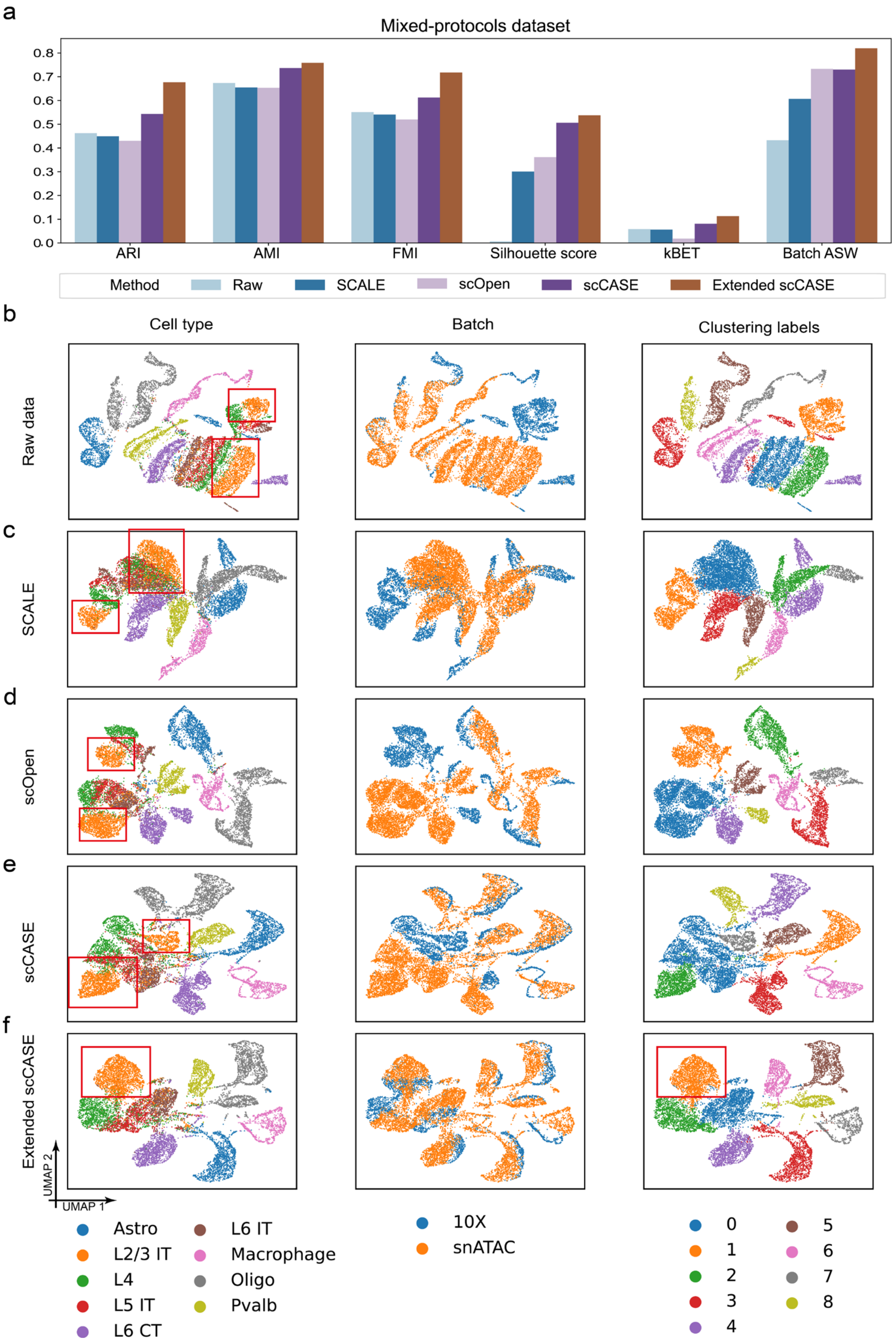

Supplementary Figure S21. Performance of scCASE and baseline methods in both preservation of biological variation and batch mixing on the Mixed-protocols dataset. a, The values of different metrics of the raw data and the data enhanced by various methods in the Mixed-protocols dataset. **b-f,** The UMAP visualization of the raw data and the data enhanced by various methods of the Mixed-protocols dataset. Source data are provided as a Source Data file.

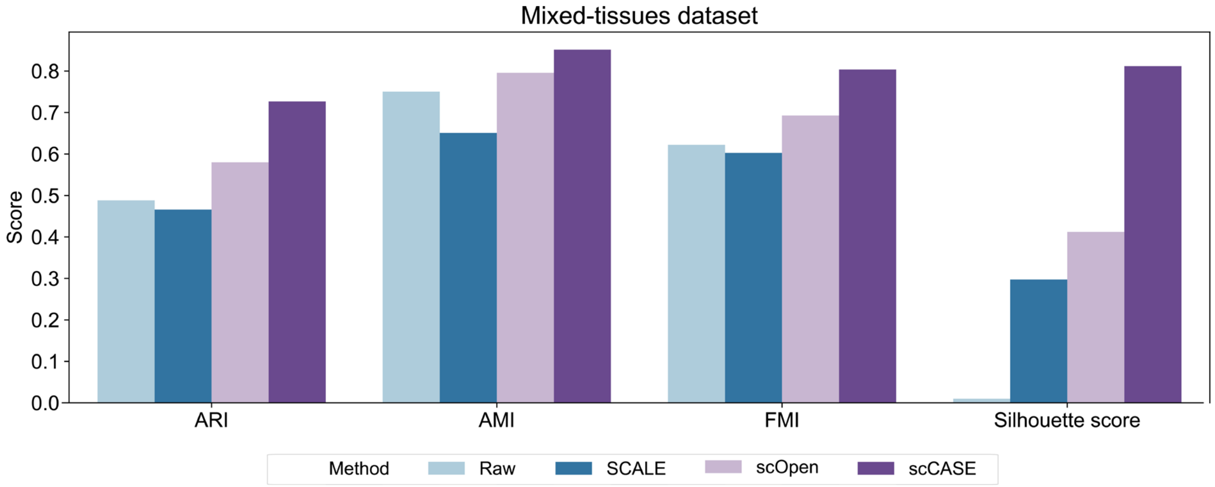

Supplementary Figure S22. Clustering performance assessed by different metrics on the Mixed-tissues dataset. Source data are provided as a Source Data file.

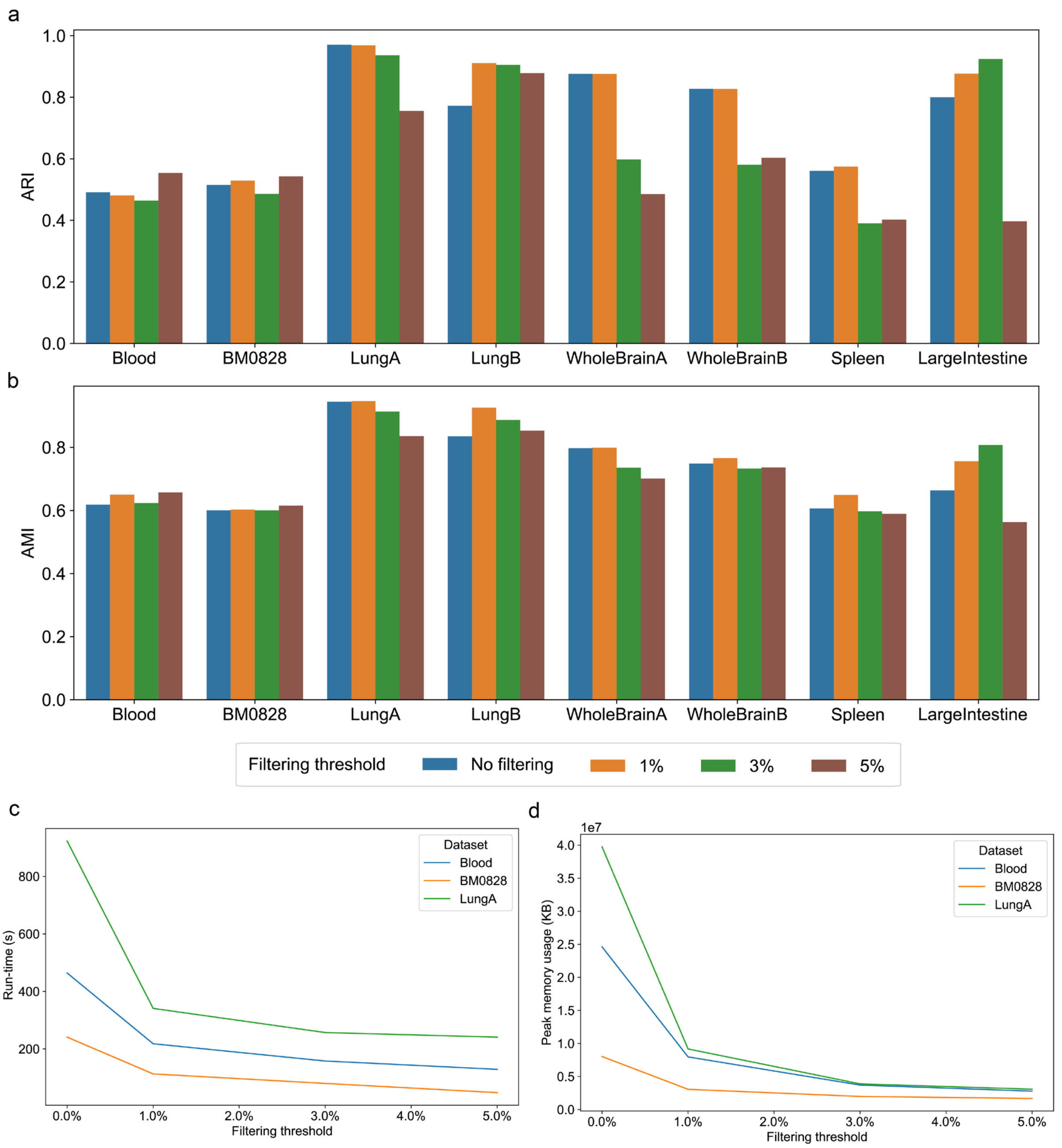

Supplementary Figure S23. The analyses of different peak filtering strategies. a, The ARI of clustering with varying peak filter threshold. **b,** The AMI of clustering with varying peak filter threshold. **c,** The run-time with varying peak filter threshold. **d,** The peak memory usage with varying peak filter threshold. Source data are provided as a Source Data file.

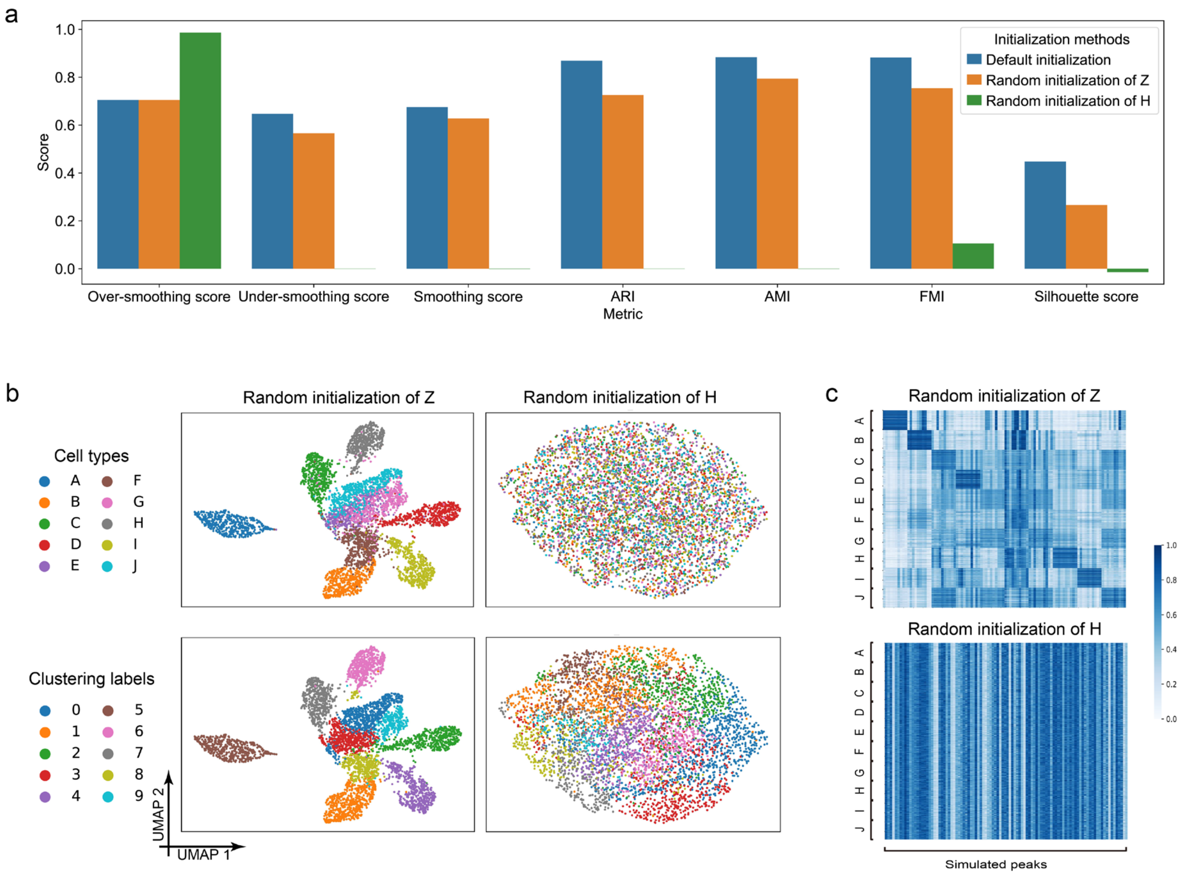

Supplementary Figure S24. Performance of scCASE with different initialization. a, The enhancement performance of different initialization. **b,** UMAP visualization of data enhanced by scCASE with random initialization of **W** and **H**. c, Cell-peak heatmap of data enhanced by scCASE with random initialization of **W** and **H**. Source data are provided as a Source Data file.

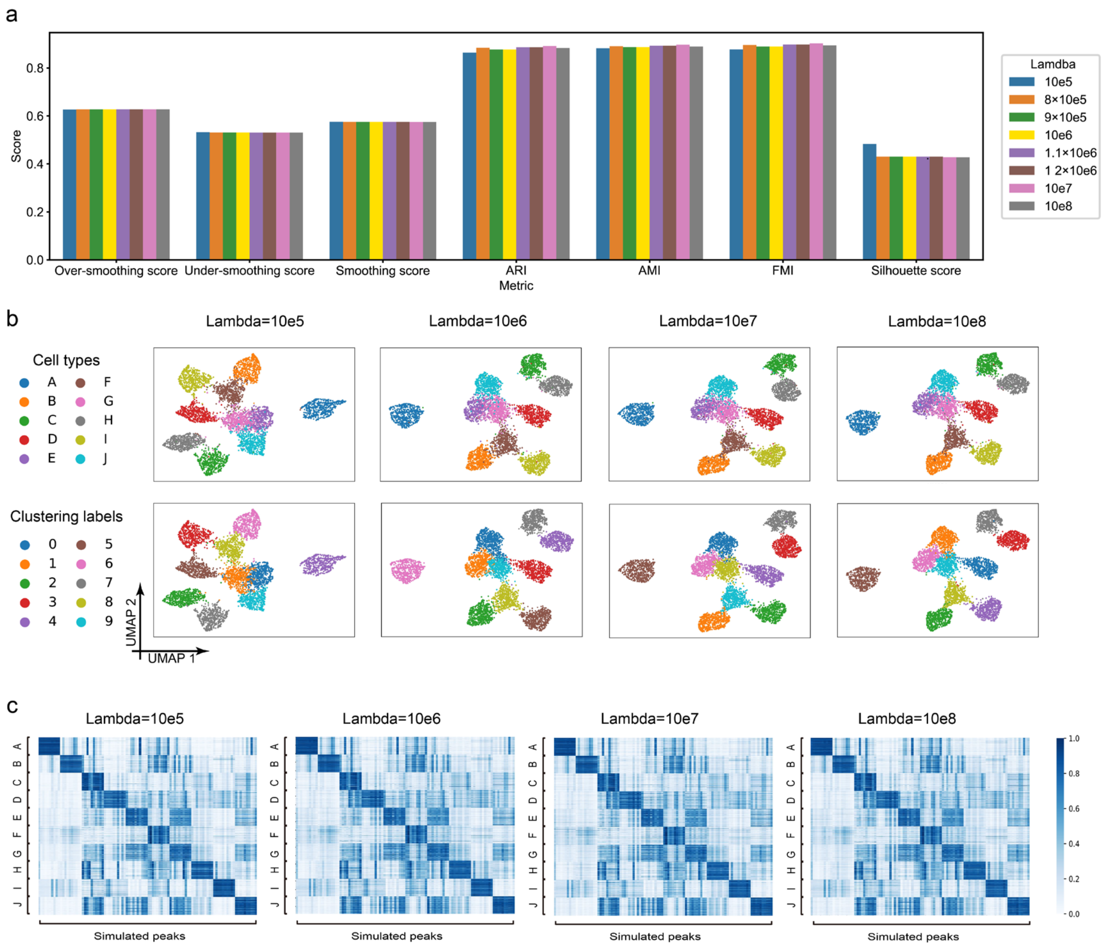

Supplementary Figure S25. Performance of scCASE with varying hyperparameter Lambda. a, The enhancement performance of scCASE with varying hyperparameter Lambda. **b,** UMAP visualization of data enhanced by scCASE with varying hyperparameter Lambda. **c,** Cell-peak heatmap of the data enhanced by scCASE with varying hyperparameter Lambda. Source data are provided as a Source Data file.

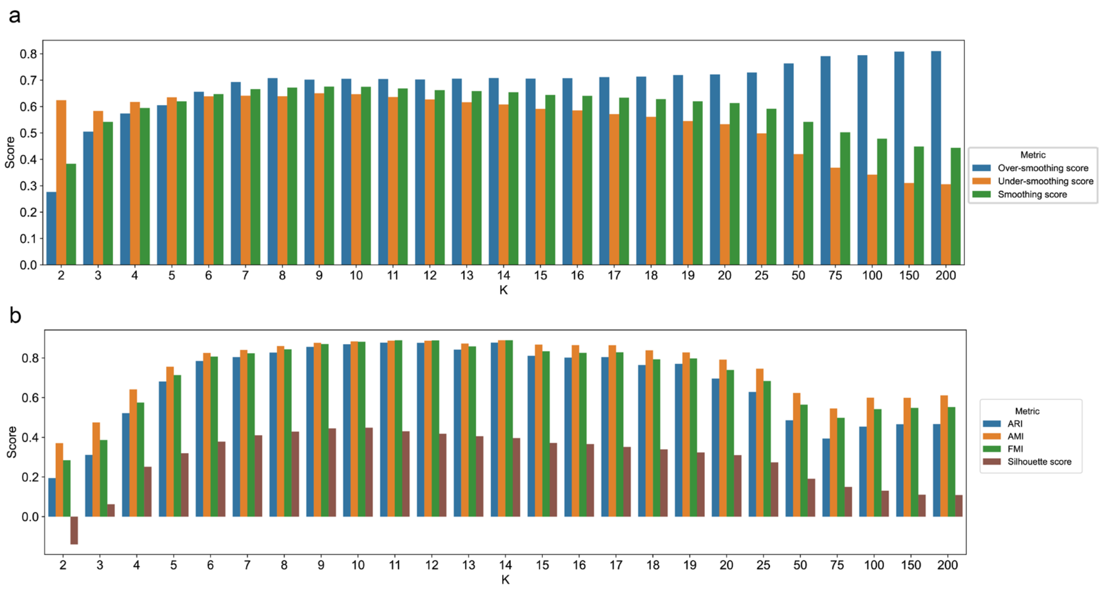

Supplementary Figure S26. Performance of scCASE with varying hyperparameter K. a, The bar plot of over-smoothing score, under-smoothing score and smoothing score of scCASE with varying hyperparameter K. **b,** The bar plot of ARI, AMI, FMI and Silhouette score of scCASE with varying hyperparameter K. Source data are provided as a Source Data file.

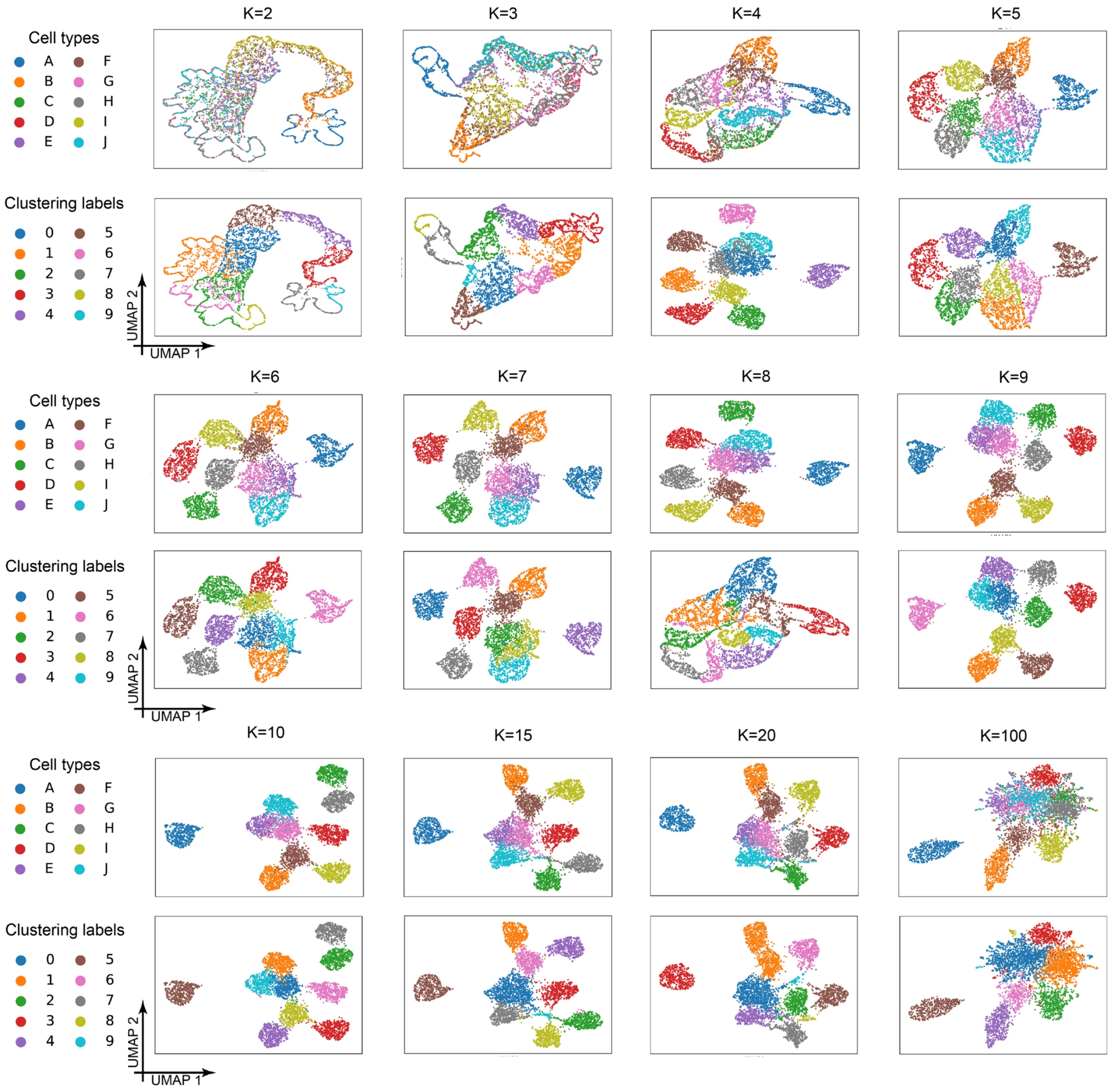

Supplementary Figure S27. UMAP visualization of the data enhanced by scCASE with varying hyperparameter *K*.

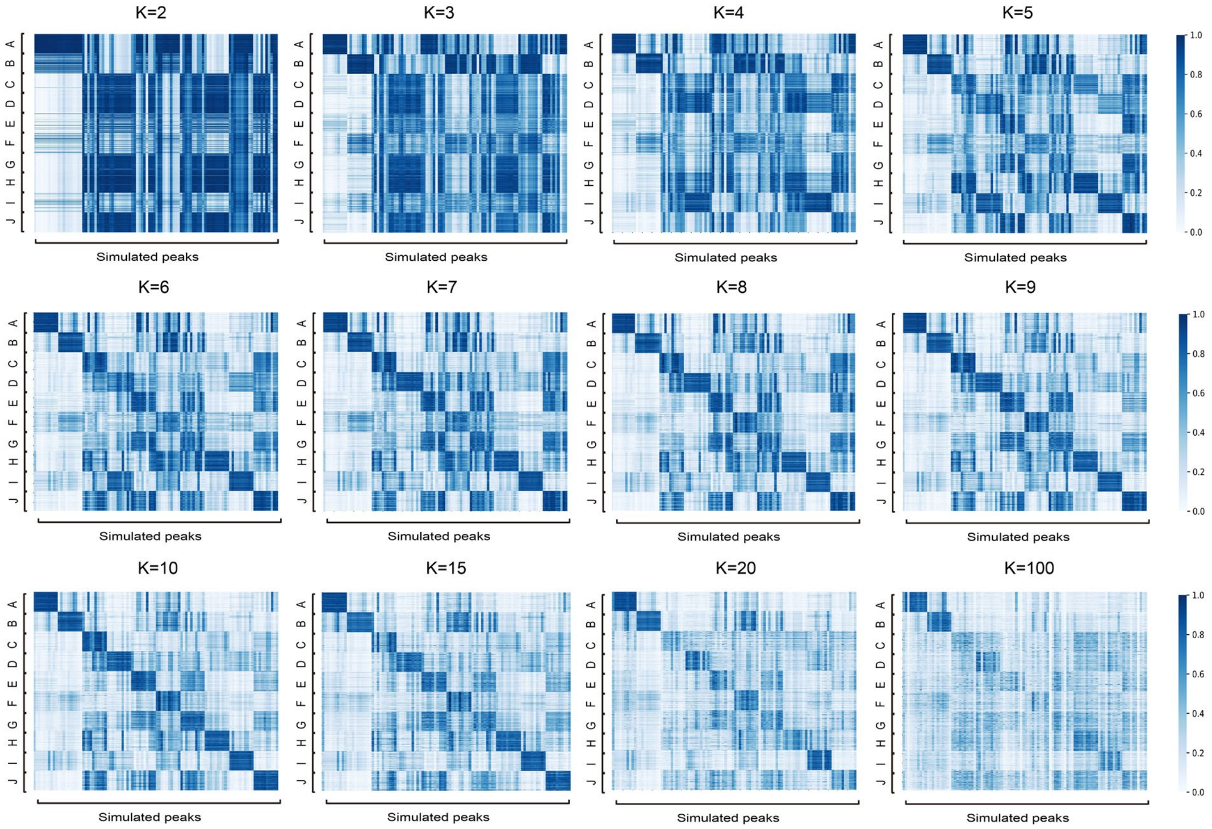

Supplementary Figure S28. Cell-peak heatmap of the data enhanced by scCASE with varying hyperparameter $\boldsymbol{K}$**.**

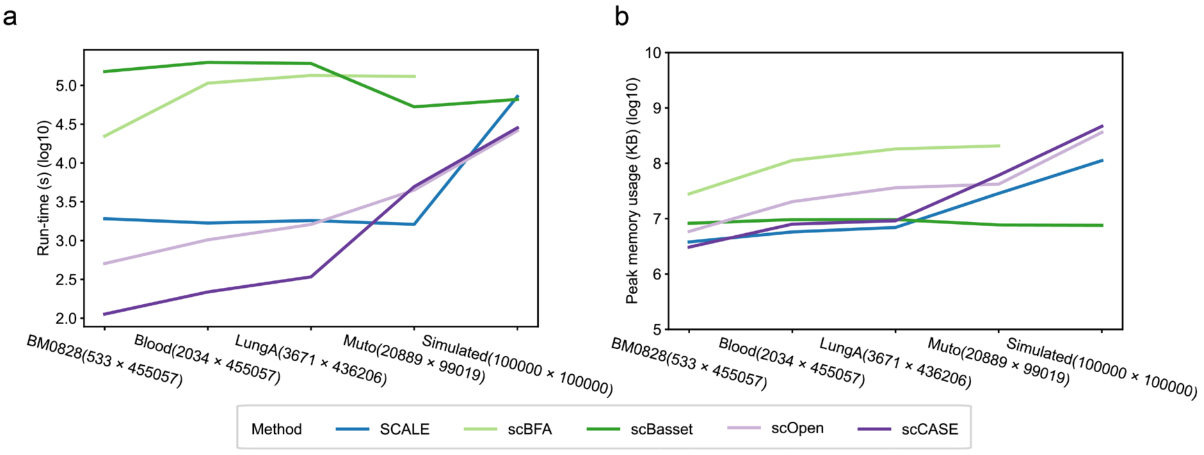

Supplementary Figure S29. Run-time and peak memory usage of scCASE and baseline methods. a, Run-time of scCASE and baseline methods. **b,** Peak memory usage of scCASE and baseline methods. Source data are provided as a Source Data file.

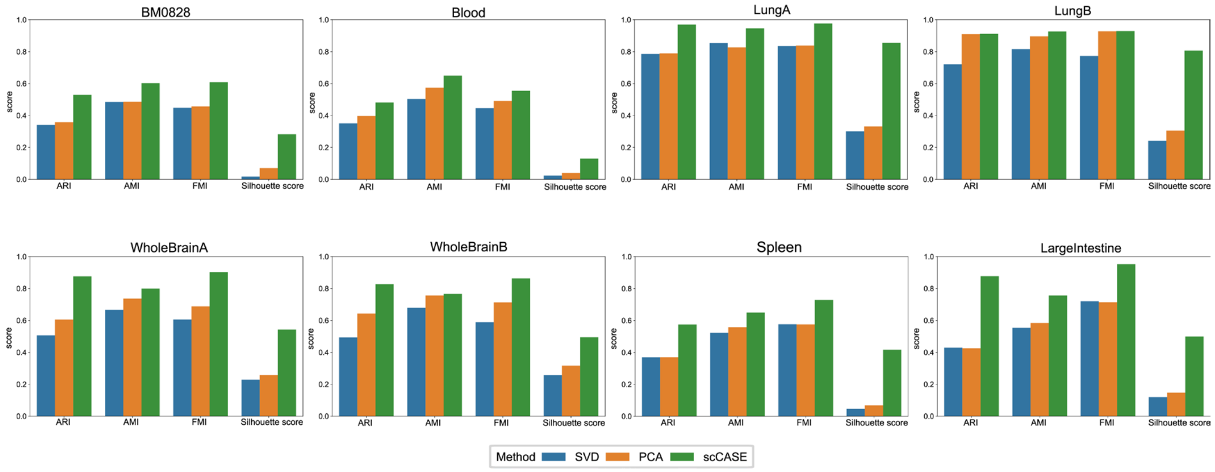

Supplementary Figure S30. Clustering performance of the data enhanced by PCA, SVD and scCASE. Source data are provided as a Source Data file.

Supplementary Tables

Supplementary Table S1. Summary of datasets used in this study.

Dataset	Species	Tissue	No. of cells	No. of peaks	No. of cell types	Sparsity (%)	Imbalance degree
Simulated	None	None	2,500	15,000	5	97.51	0.000
Blood	*Homo sapiens*	Bone marrow	2,034	430,107	10	98.97	0.106
BM0828	*Homo sapiens*	Bone marrow	533	320,083	7	98.66	0.024
WholeBrainA	*Mus musculus*	Whole brain	4,821	436,067	8	98.51	0.146
WholeBrainB	*Mus musculus*	Whole brain	2,880	436,019	8	98.28	0.124
LungA	*Mus musculus*	Lung	3,671	431,903	6	99.14	0.110
LungB	*Mus musculus*	Lung	3,381	434,037	7	99.09	0.085
Spleen	*Mus musculus*	Spleen	3,674	418,818	6	98.75	0.310
LargeIntestine	*Mus musculus*	LargeIntestine	1,670	434,913	3	99.01	0.378
Muto	*Homo sapiens*	Kidney	20,889	99,019	5	93.51	0.214
PBMC	*Homo sapiens*	PMBC	8,483	107,194	10	93.19	0.133
Mixed-tissues	*Mus musculus*	Lung and Spleen	7,117	435,206	7	98.98	0.205
Mixed-protocols	*Mus musculus*	Brain	11,755	478,615	9	98.93	0.027

Supplementary Table S2. The complete results of clustering metrics.

Dataset	Methods	ARI	AMI	FMI	Silhouette score
Blood	RAW	0.31689	0.51359	0.41325	0.00289
Blood	scCASE	0.48079	0.65007	0.55464	0.13047
Blood	scCASER	0.54965	0.65433	0.61621	0.13709
Blood	SCALE	0.30904	0.50289	0.40903	0.03749
Blood	scBasset	0.38821	0.59775	0.47848	0.09697
Blood	scBFA	0.39076	0.60157	0.47599	0.04432
Blood	scOpen	0.42746	0.62089	0.50785	0.11448
BM0828	RAW	0.45160	0.57695	0.53530	0.00247
BM0828	scCASE	0.52911	0.60264	0.60911	0.28181
BM0828	scCASER	0.53534	0.63571	0.62549	0.32696
BM0828	SCALE	0.43223	0.57292	0.51934	0.14010
BM0828	scBasset	0.50574	0.62489	0.58286	0.27605
BM0828	scBFA	0.47290	0.58177	0.55676	0.11981
BM0828	scOpen	0.51566	0.63388	0.59110	0.27096
LargeIntestine	RAW	0.40664	0.56671	0.70170	0.00093
LargeIntestine	scCASE	0.87682	0.75638	0.95277	0.49937
LargeIntestine	scCASER	0.95515	0.85775	0.98277	0.55331
LargeIntestine	SCALE	0.42951	0.52055	0.72159	0.16647
LargeIntestine	scBFA	0.41550	0.55576	0.70851	0.15687
LargeIntestine	scOpen	0.42144	0.58529	0.71079	0.44203
LargeIntestine	scBasset	0.87428	0.76569	0.95441	0.24021
LungA	RAW	0.74001	0.83199	0.79969	0.00964
LungA	scCASE	0.96890	0.94689	0.97619	0.85500
LungA	scCASER	0.97096	0.94777	0.97777	0.86005
LungA	SCALE	0.68031	0.76267	0.75195	0.49799
LungA	scBasset	0.88167	0.88720	0.90908	0.63079
LungA	scBFA	0.87451	0.87666	0.90353	0.53580
LungA	scOpen	0.88798	0.90112	0.91395	0.60595

Supplementary Table S2 (continue). The complete results of clustering metrics.

**Dataset**	**Methods**	**ARI**	**AMI**	**FMI**	**Silhouette score**
LungB	RAW	0.69647	0.81011	0.75270	0.00844
LungB	scCASE	0.91111	0.92613	0.92842	0.80709
LungB	scCASER	0.97106	0.95575	0.97663	0.81376
LungB	SCALE	0.71527	0.77003	0.76838	0.39604
LungB	scBasset	0.88769	0.88628	0.90928	0.61081
LungB	scBFA	0.84788	0.84529	0.87674	0.39584
LungB	scOpen	0.90557	0.91409	0.92375	0.54335
Spleen	RAW	0.33659	0.50610	0.54915	0.00125
Spleen	scCASE	0.57480	0.64945	0.72827	0.41662
Spleen	scCASER	0.79280	0.69529	0.87948	0.38029
Spleen	SCALE	0.40622	0.52973	0.60615	0.08148
Spleen	scBasset	0.35633	0.58666	0.56467	0.24385
Spleen	scBFA	0.35840	0.58473	0.56656	0.11999
Spleen	scOpen	0.50590	0.67883	0.67817	0.24215
WholeBrainA	RAW	0.47340	0.66188	0.58001	0.00547
WholeBrainA	scCASE	0.87556	0.79912	0.90339	0.54260
WholeBrainA	scCASER	0.85408	0.77655	0.88657	0.51095
WholeBrainA	SCALE	0.41703	0.61403	0.53099	0.42473
WholeBrainA	scBasset	0.55598	0.72974	0.64779	0.44564
WholeBrainA	scBFA	0.59930	0.73347	0.68284	0.39359
WholeBrainA	scOpen	0.55374	0.74586	0.64592	0.44481
WholeBrainB	RAW	0.50206	0.66301	0.59686	0.00541
WholeBrainB	scCASE	0.82731	0.76608	0.86350	0.49407
WholeBrainB	scCASER	0.87415	0.80657	0.90159	0.52757
WholeBrainB	SCALE	0.50945	0.67275	0.60288	0.43883
WholeBrainB	scBasset	0.54118	0.73944	0.62990	0.46615
WholeBrainB	scBFA	0.65468	0.74723	0.72272	0.34705
WholeBrainB	scOpen	0.53118	0.74582	0.62112	0.44110

Supplementary Table S3. Identified significant pathways in the GREAT analysis by the peaks obtained from monocytes in the Blood dataset with scCASE.

**Term name**	**Binom raw *P*-Val[1]**	**Binom FDR *Q*-Val[2]**
response to bacterium	4.00E-09	5.26E-05
response to lipopolysaccharide	4.85E-09	3.19E-05
response to molecule of bacterial origin	7.69E-09	3.37E-05
positive regulation of immune system process	2.38E-08	7.83E-05
regulation of immune system process	4.99E-08	1.31E-04
response to other organism	1.59E-07	3.49E-04
response to external biotic stimulus	1.81E-07	3.41E-04
cell chemotaxis	2.25E-07	3.69E-04
response to oxygen-containing compound	2.89E-07	4.22E-04
response to biotic stimulus	4.69E-07	6.16E-04
response to external stimulus	5.08E-07	6.07E-04
immune system process	9.46E-07	1.04E-03
positive regulation of response to stimulus	1.00E-06	1.02E-03
regulation of immune response	1.21E-06	1.14E-03
response to lipid	1.80E-06	1.58E-03
surfactant homeostasis	4.67E-06	3.61E-03
chemical homeostasis within a tissue	8.84E-06	5.53E-03
cellular response to lipopolysaccharide	1.17E-05	7.01E-03
regulation of innate immune response	1.18E-05	6.75E-03
regulation of response to stress	1.30E-05	7.12E-03

1. **Binom raw *P*-Val** means the uncorrected one-sided *p*-value from the binomial test over genomic egions.
2. **Binom FDR *Q*-Val** means the false discovery rate corrected (FDR-corrected) **Binom raw *P*-Val** namely *q*-value.

Supplementary Table S4. Identified significant pathways in the GREAT analysis by the peaks obtained from T cells in the LungB dataset with scCASE.

**Term name**	**Binom raw *P*-Val**[1]	**Binom FDR *Q*-Val**[2]
abnormal T cell physiology	1.90E-13	1.74E-09
abnormal CD8-positive, alpha beta T cell morphology	1.13E-12	5.19E-09
abnormal T cell activation	1.47E-12	4.50E-09
abnormal CD8-positive, alpha-beta T cell number	1.64E-12	3.75E-09
abnormal T cell proliferation	2.65E-11	4.86E-08
abnormal lymphocyte physiology	6.75E-11	1.03E-07
abnormal leukocyte physiology	2.58E-10	3.38E-07
abnormal immune cell physiology	3.07E-10	3.52E-07
abnormal cell-mediated immunity	3.48E-10	3.54E-07
abnormal adaptive immunity	5.79E-10	5.31E-07
abnormal alpha-beta T cell morphology	5.82E-10	4.84E-07
abnormal alpha-beta T cell number	8.90E-10	6.79E-07
abnormal leukopoiesis	9.17E-10	6.46E-07
abnormal effector T cell morphology	1.55E-09	1.02E-06
abnormal blood cell physiology	1.77E-09	1.08E-06
abnormal hematopoietic system physiology	2.17E-09	1.24E-06
abnormal CD4-positive, alpha beta T cell number	2.49E-09	1.34E-06
increased T cell number	2.76E-09	1.41E-06
abnormal CD4-positive, alpha beta T cellmorphology	3.00E-09	1.45E-06
abnormal immune system physiology	9.96E-09	4.56E-06

1. **Binom raw *P*-Val** means the uncorrected one-sided *p*-value from the binomial test over genomic egions.
2. **Binom FDR *Q*-Val** means the false discovery rate corrected (FDR-corrected) **Binom raw *P*-Val** namely *q*-value.

Supplementary Table S5. The genes correspond to the cell type-specific peaks identified by scCASE.

**Dataset**	**Cell**	**Region**	**Gene symbol**	**Reference**
LungA	Monocytes	chr2:219,246,758-219,247,258	*SLC11A1*	UniProt UCSC
LungA	Monocytes	chr6:41,239,630-41,240,130	*TREM1*	UniProt UCSC
LungA	Monocytes	chr20:30,622,298-30,622,798	*HCK*	UniProt UCSC
LungA	Monocytes	chr17:38,249,037-38,256,978	*NR1D1*	UniProt UCSC
Blood	T cells	chr6:124,834,895-124,836,445	*Cd4*	UniProt UCSC
Blood	T cells	chr9:44,806,826-44,817,673	*Cd3e*	UniProt UCSC
Blood	T cells	chr7:25,154,610-25,155,352	*Kcnn4*	UniProt UCSC
Blood	T cells	chr12:114,092,788-114,093,703	*Gpr132(G2A)*	UniProt UCSC
Blood	T cells	chr11:46,201,573-46,203,658	*Itk*	UniProt UCSC
Blood	T cells	chr11:46,157,147-46,158,328	*Itk*	UniProt UCSC

Supplementary Table S6. The identified blood-associated TF binding motifs.

**TF binding motifs**	**Reference**
JUNB	Santaguida, M. T., et al.[19]
BATF	Wang, J., et al.[20]
NFE2	Hung, H., et al.[21]
GATA1	Calligaris, R., et al.[22]
TAL1	Aplan, P. D., et al.[23]
GATA2	Bresnick, E. H., Mabel M. J., and Koichi R. K.[24]
SPI1	Le Coz, C., et al.[25]
SPIC	UniProt
ETV6	Hock, H., and Akiko S..[26]
IRF7	Ning, S., J. S. Pagano, and G. N. Barber.[27]
IRF8	Salem, S., David S., and Philippe Gros.[28]
SPIB	Schotte, R., et al.[29]
RUNX3	Menezes, A., et al.[30]
HLF	Komorowska, K., et al.[31]
CEBPA	Sierra, J., and Josep F. Nomdedeu.[32]
CEBPD	Spek, C. A., et al.[33]
CEBPG	Jiang, Y., et al.[34]
CEBPB	Yokota, A., et al.[35]
CEBPE	Lekstrom-Himes, J. A., et al.[36]
NFE2	Rost, M. S., et al.[37]
CTCF	Herold, M., Marek B., and Rainer R.[38]
SNAI2	Nerlov, C., and Thomas G.[39]
ID4	Martin, C. H., et al.[40]
TCF3	Somasundaram, R., Prasad, M. A., Ungerb¨ack, J. and Sigvardsson, M.[41]
TCF4	Somasundaram, R., Prasad, M. A., Ungerb¨ack, J. and Sigvardsson, M.[41]

Supplementary Table S6 (continue). The identified blood-associated TF binding motifs.

TF binding motifs	Reference
FIGLA	Virant-Klun, I.[42]
ZEB1	Schep, A. N., Wu, B., Buenrostro, J. D. and Greenleaf, W. J. [43]
FOS::JUN	Liebermann, D., Gregory, B. and Hoffman, B.[44]
FOS	Shafarenko, M., Amanullah, A., Gregory, B., Liebermann, D. A. and Hoffman, B.[45]
JUND	Liebermann, D. A. and Hoffman, B.[46]
ELF5	Yamamizu, K. et al.[47]<br>Mabbott, N. A., Baillie, J. K., Hume, D. A. and Freeman, T. C.[48]
PKNOX2	Cagnan. et al.[49]
PKNOX1	Di Rosa, P. et al.[50]
TGIF2	Sugimura, R. et al.[51]
ATF4	Zhao, Y. et al.[52]
JDP2	Ji, H. et al.[53]

Supplementary References

1. Buttner, M., Miao, Z., Wolf, F.A., Teichmann, S.A. & Theis, F.J. A test metric for assessing single-cell RNA-seq batch correction. *Nat. Methods* **16**, 43-49 (2019).

2. Luecken, M.D. et al. Benchmarking atlas-level data integration in single-cell genomics. *Nat. Methods* **19**, 41-50 (2022).

3. Li, Z. et al. Chromatin-accessibility estimation from single-cell ATAC-seq data with scOpen. *Nat. Commun.* **12**, 6386 (2021).

4. Chen, S. et al. RA3 is a reference-guided approach for epigenetic characterization of single cells. *Nat. Commun.* **12**, 2177 (2021).

5. Liu, Q., Chen, S., Jiang, R. & Wong, W.H. Simultaneous deep generative modelling and clustering of single-cell genomic data. *Nat. Mach. Intell.* **3**, 536-544 (2021).

6. Cusanovich, D.A. et al. A Single-Cell Atlas of In Vivo Mammalian Chromatin Accessibility. *Cell* **174**, 1309-1324 e1318 (2018).

7. King, K.Y. & Goodell, M.A. Inflammatory modulation of HSCs: viewing the HSC as a foundation for the immune response. *Nat. Rev. Immunol.* **11**, 685-692 (2011).

8. Rodrigues, N.P. et al. GATA-2 regulates granulocyte-macrophage progenitor cell function. *Blood* **112**, 4862-4873 (2008).

9. Buenrostro, J.D. et al. Integrated Single-Cell Analysis Maps the Continuous Regulatory Landscape of Human Hematopoietic Differentiation. *Cell* **173**, 1535-1548 e1516 (2018).

10. Xiong, L. et al. SCALE method for single-cell ATAC-seq analysis via latent feature extraction. *Nat. Commun.* **10**, 4576 (2019).

11. Chen, S., Wang, R., Long, W. & Jiang, R. ASTER: accurately estimating the number of cell types in single-cell chromatin accessibility data. *Bioinformatics* **39** (2023).

12. Xiong, L. et al. Online single-cell data integration through projecting heterogeneous datasets into a common cell-embedding space. *Nat. Commun.* **13**, 6118 (2022).

13. Yuan, H. & Kelley, D.R. scBasset: sequence-based modeling of single-cell ATAC-seq using convolutional neural networks. *Nat. Methods* **19**, 1088-1096 (2022).

14. Liu, Y., Zhang, J., Wang, S., Zeng, X. & Zhang, W. Are dropout imputation methods for scRNA-seq effective for scATAC-seq data? *Brief. Bioinformatics* **23**, bbab442 (2022).

15. Lin, X. & Boutros, P.C. Optimization and expansion of non-negative matrix factorization. *BMC Bioinform.* **21**, 7 (2020).

16. Chen, L., Xu, J. & Li, S.C. DeepMF: Deciphering the latent patterns in omics profiles with a deep learning method. *BMC Bioinform.* **20**, 1-13 (2019).

17. Wan, X., Zhang, B., Zou, G. & Chang, F. Sparse data recommendation by fusing continuous imputation denoising autoencoder and neural matrix factorization. *Appl. Sci.* **9**, 54 (2018).

18. Dhont, M., Tsiporkova, E. & González-Deleito, N. Deriving spatio-temporal trajectory fingerprints from mobility data using non-negative matrix factorisation. In *2021 International Conference on Data Mining Workshops* 750-759 (2021).

19. Santaguida, M.T., Schepers, K., King, B.C. & Passegue, E. JunB Limits Hematopoietic Stem Cell (HSC) Functions as a Protective Mechanism against Initiation of Myeloid Malignancy. *Blood* **112**, 1358 (2008).

20. Wang, J. et al. A differentiation checkpoint limits hematopoietic stem cell self-renewal in response to DNA damage. *Cell* **148**, 1001-1014 (2012).

21. Hung, H.-L., Kim, A.Y., Hong, W., Rakowski, C. & Blobel, G.A. Stimulation of NF-E2 DNA binding by CREB-binding protein (CBP)-mediated acetylation. *J. Biol. Chem.* **276**, 10715-10721 (2001).

22. Calligaris, R., Bottardi, S., Cogoi, S., Apezteguia, I. & Santoro, C. Alternative translation initiation site usage results in two functionally distinct forms of the GATA-1 transcription factor. *Proc. Natl. Acad. Sci. U.S.A.* **92**, 11598-11602 (1995).

23. Aplan, P., Nakahara, K., Orkin, S.H. & Kirsch, I.R. The SCL gene product: a positive regulator of erythroid differentiation. *Embo J.* **11**, 4073-4081 (1992).

24. Bresnick, E.H., Jung, M.M. & Katsumura, K.R. Human GATA2 mutations and hematologic disease: how many paths to pathogenesis? *Blood Adv.* **4**, 4584-4592 (2020).

25. Le Coz, C. et al. Constrained chromatin accessibility in PU. 1-mutated agammaglobulinemia patients. *J. Exp. Med.* **218**, e20201750 (2021).

26. Hock, H. & Shimamura, A. ETV6 in hematopoiesis and leukemia predisposition. *Semin. Hematol.* **54**, 98-104 (2017).

27. Ning, S., Pagano, J. & Barber, G. IRF7: activation, regulation, modification and function. *Genes Immun.* **12**, 399-414 (2011).

28. Salem, S., Salem, D. & Gros, P. Role of IRF8 in immune cells functions, protection against infections, and susceptibility to inflammatory diseases. *Hum. Genet.* **139**, 707-721 (2020).

29. Schotte, R., Nagasawa, M., Weijer, K., Spits, H. & Blom, B. The ETS transcription factor Spi-B is required for human plasmacytoid dendritic cell development. *J. Exp. Med.* **200**, 1503-1509 (2004).

30. Menezes, A.C. et al. RUNX3 overexpression inhibits normal human erythroid development. *Sci. Rep.* **12**, 1243 (2022).

31. Komorowska, K. et al. Hepatic leukemia factor maintains quiescence of hematopoietic stem cells and protects the stem cell pool during regeneration. *Cell Rep.* **21**, 3514-3523 (2017).

32. Sierra, J. & Nomdedeu, J.F. CEBPA bZip mutations: just a single shot. *Blood* **138**, 1091-1092 (2021).

33. Spek, C.A., Aberson, H.L., Butler, J.M., de Vos, A.F. & Duitman, J. CEBPD potentiates the macrophage inflammatory response but CEBPD knock-out macrophages fail to identify CEBPD-dependent pro-inflammatory transcriptional programs. *Cells* **10**, 2233 (2021).

34. Jiang, Y. et al. CEBPG promotes acute myeloid leukemia progression by enhancing EIF4EBP1. *Cancer Cell Int.* **21**, 1-12 (2021).

35. Yokota, A. et al. C/EBPβ is a critical mediator of IFN-α–induced exhaustion of chronic myeloid leukemia stem cells. *Blood Adv.* **3**, 476-488 (2019).

36. Lekstrom-Himes, J.A., Dorman, S.E., Kopar, P., Holland, S.M. & Gallin, J.I. Neutrophil-specific granule deficiency results from a novel mutation with loss of function of the transcription factor CCAAT/enhancer binding protein ε. *J. Exp. Med.* **189**, 1847-1852 (1999).

37. Rost, M.S. et al. Nfe2 is dispensable for early but required for adult thrombocyte formation and function in zebrafish. *Blood Adv.* **2**, 3418-3427 (2018).

38. Herold, M., Bartkuhn, M. & Renkawitz, R. CTCF: insights into insulator function during development. *Development* **139**, 1045-1057 (2012).

39. Nerlov, C. & Graf, T. PU. 1 induces myeloid lineage commitment in multipotent hematopoietic progenitors. *Genes Dev.* **12**, 2403-2412 (1998).

40. Martin, C.H., Woll, P.S., Ni, Z., Zúñiga-Pflücker, J.C. & Kaufman, D.S. Differences in lymphocyte developmental potential between human embryonic stem cell and umbilical cord blood–derived hematopoietic progenitor cells. *Blood* **112**, 2730-2737 (2008).

41. Somasundaram, R., Prasad, M.A., Ungerbäck, J. & Sigvardsson, M. Transcription factor networks in B-cell differentiation link development to acute lymphoid leukemia. *Blood* **126**, 144-152 (2015).

42. Virant-Klun, I. Very small embryonic-like stem cells: a potential developmental link between germinal lineage and hematopoiesis in humans. *Stem Cells Dev.* **25**, 101-113 (2016).

43. Schep, A.N., Wu, B., Buenrostro, J.D. & Greenleaf, W.J. chromVAR: inferring transcription-factor-associated accessibility from single-cell epigenomic data. *Nat. Methods* **14**, 975-978 (2017).

44. Liebermann, D., Gregory, B. & Hoffman, B. AP-1 (Fos/Jun) transcription factors in hematopoietic differentiation and apoptosis. *Int. J. Oncol.* **12**, 685-1385 (1998).

45. Shafarenko, M., Amanullah, A., Gregory, B., Liebermann, D.A. & Hoffman, B. Fos modulates myeloid cell survival and differentiation and partially abrogates the c-Myc block in terminal myeloid differentiation. *Blood* **103**, 4259-4267 (2004).

46. Liebermann, D.A. & Hoffman, B. Myeloid differentiation (MyD) primary response genes in hematopoiesis. *Oncogene* **21**, 3391-3402 (2002).

47. Yamamizu, K. et al. Identification of transcription factors for lineage-specific ESC differentiation. *Stem Cell Rep.* **1**, 545-559 (2013).

48. Mabbott, N.A., Baillie, J.K., Hume, D.A. & Freeman, T.C. Meta-analysis of lineage-specific gene expression signatures in mouse leukocyte populations. *Immunobiology* **215**, 724-736 (2010).

49. Cagnan, I., Cosgun, E., Konu, O., Uckan, D. & Gunel-Ozcan, A. PKNOX2 expression and regulation in the bone marrow mesenchymal stem cells of Fanconi anemia patients and healthy donors. *Mol. Biol. Rep.* **46**, 669-678 (2019).

50. Di Rosa, P. et al. The homeodomain transcription factor Prep1 (pKnox1) is required for hematopoietic stem and progenitor cell activity. *Dev. Biol.* **311**, 324-334 (2007).

51. Sugimura, R. et al. Haematopoietic stem and progenitor cells from human pluripotent stem cells. *Nature* **545**, 432-438 (2017).

52. Zhao, Y. et al. ATF4 plays a pivotal role in the development of functional hematopoietic stem cells in mouse fetal liver. *Blood* **126**, 2383-2391 (2015).

53. Ji, H. et al. Comprehensive methylome map of lineage commitment from haematopoietic progenitors. *Nature* **467**, 338-342 (2010).
