# Supplementary material for: scCASE: accurate and interpretable enhancement for single-cell chromatin accessibility sequencing data
Source: Nat Commun. 2024 Feb 22;15:1629. doi: 10.1038/s41467-024-46045-w (PMC10884038; doi:10.1038/s41467-024-46045-w)
Supplement: Supplementary file 3 — Reporting Summary [file 41467_2024_46045_MOESM3_ESM.pdf]

nature portfolio

Corresponding author(s): Shengquan Chen

Last updated by author(s): Feb 5, 2024

Reporting Summary

Nature Portfolio wishes to improve the reproducibility of the work that we publish. This form provides structure for consistency and transparency in reporting. For further information on Nature Portfolio policies, see our Editorial Policies and the Editorial Policy Checklist.

Statistics

For all statistical analyses, confirm that the following items are present in the figure legend, table legend, main text, or Methods section.

n/a	Confirmed	
☐	☒	The exact sample size (*n*) for each experimental group/condition, given as a discrete number and unit of measurement
☐	☒	A statement on whether measurements were taken from distinct samples or whether the same sample was measured repeatedly
☐	☒	The statistical test(s) used AND whether they are one- or two-sided<br>*Only common tests should be described solely by name; describe more complex techniques in the Methods section.*
☐	☒	A description of all covariates tested
☐	☒	A description of any assumptions or corrections, such as tests of normality and adjustment for multiple comparisons
☐	☒	A full description of the statistical parameters including central tendency (e.g. means) or other basic estimates (e.g. regression coefficient) AND variation (e.g. standard deviation) or associated estimates of uncertainty (e.g. confidence intervals)
☐	☒	For null hypothesis testing, the test statistic (e.g. *F*, *t*, *r*) with confidence intervals, effect sizes, degrees of freedom and *P* value noted<br>*Give P values as exact values whenever suitable.*
☐	☒	For Bayesian analysis, information on the choice of priors and Markov chain Monte Carlo settings
☒	☐	For hierarchical and complex designs, identification of the appropriate level for tests and full reporting of outcomes
☐	☒	Estimates of effect sizes (e.g. Cohen's *d*, Pearson's *r*), indicating how they were calculated

Our web collection on statistics for biologists contains articles on many of the points above.

Software and code

Policy information about availability of computer code

Data collection	The study does not involve data collection. No software was used for data collection. We used publicly available datasets to evaluate the performance of our method.

Data analysis	scanpy(1.9.1), https://github.com/scverse/scanpy<br>episcanpy(0.3.2), https://github.com/colomemaria/epiScanpy<br>scikit-learn(1.1.2), https://github.com/scikit-learn/scikit-learn<br>scipy(1.9.0), https://github.com/scipy/scipy<br>epiaster(0.0.2), https://github.com/BioX-NKU/ASTER<br>SCALE(1.1.2), https://github.com/jsxlei/SCALE<br>scBFA(1.0), https://github.com/quon-titative-biology/scBFA<br>scBasset(0.1), https://github.com/calico/scBasset<br>scOpen(0.1.8), https://github.com/CostaLab/scopen<br>seaborn(0.11.2), https://github.com/mwaskom/seaborn<br>Great(4.0.4), http://great.stanford.edu/public/html<br>chromVAR(0.99.1), https://github.com/GreenleafLab/chromVAR<br>simCAS(1.0.0), https://github.com/Chen-Li-17/simCAS<br>snpsea(1.0.2), https://github.com/slowkow/snpsea<br>scCASE is availible at https://github.com/BioX-NKU/scCASE<br>codes and data for reproducing the results of scCASE is availible at https://zenodo.org/records/8382876

For manuscripts utilizing custom algorithms or software that are central to the research but not yet described in published literature, software must be made available to editors and reviewers. We strongly encourage code deposition in a community repository (e.g. GitHub). See the Nature Portfolio guidelines for submitting code & software for further information.

Data

Policy information about availability of data

All manuscripts must include a data availability statement. This statement should provide the following information, where applicable:

- Accession codes, unique identifiers, or web links for publicly available datasets
- A description of any restrictions on data availability
- For clinical datasets or third party data, please ensure that the statement adheres to our policy

The Blood and BM0828 datasets and their corresponding bulk data can be retrieved from NCBI Gene Expression Omnibus (GEO) with accession number GSE96772. The datasets of various mouse tissues are available at https://atlas.gs.washington.edu/mouse-atac/data. The Muto dataset can be retrieved from GEO with accession number GSE151302. The PBMC dataset profiled by 10x Genomics via "Single Cell Multiome ATAC + Gene Expression Sequencing" can be downloaded at https://www.10xgenomics.com/resources/datasets/pbmc-from-a-healthy-donor-granulocytes-removed-through-cell-sorting-10-k-1-standard-2-0-0. The Mix-protocols dataset was concatenated from two mouse brain datasets profiled by different protocols, which are available in GEO with accession number GSE126724 and at https://support.10xgenomics.com/single-cell-atac/datasets/1.1.0/atac_v1_adult_brain_fresh_5k. UCSC Genome Browser and UniProt database are used in this study.

Research involving human participants, their data, or biological material

Policy information about studies with human participants or human data. See also policy information about sex, gender (identity/presentation), and sexual orientation and race, ethnicity and racism.

Reporting on sex and gender	N/A
Reporting on race, ethnicity, or other socially relevant groupings	N/A
Population characteristics	N/A
Recruitment	N/A
Ethics oversight	N/A

Note that full information on the approval of the study protocol must also be provided in the manuscript.

Field-specific reporting

Please select the one below that is the best fit for your research. If you are not sure, read the appropriate sections before making your selection.

☒ Life sciences ☐ Behavioural & social sciences ☐ Ecological, evolutionary & environmental sciences

For a reference copy of the document with all sections, see nature.com/documents/nr-reporting-summary-flat.pdf

Life sciences study design

All studies must disclose on these points even when the disclosure is negative.

Sample size	We conducted comprehensive comparisons on various datasets, including a simulated scCAS dataset, eight publicly available scCAS datasets, two scCAS datasets annotated base on the paired scRNA-seq data, and two mixed scCAS datasets. We utilized simCAS to create simulated dataset, and we chose sample sizes based on the default parameters. All other datasets are publicly available. No experiments in the present study.
Data exclusions	Cell types that accounted for less than three percent of the total cells were discarded along with the unknown categories to ensure evaluation credibility.
Replication	No experiments in the present study.
Randomization	No experiments in the present study.
Blinding	No experiments in the present study.

Reporting for specific materials, systems and methods

We require information from authors about some types of materials, experimental systems and methods used in many studies. Here, indicate whether each material, system or method listed is relevant to your study. If you are not sure if a list item applies to your research, read the appropriate section before selecting a response.

Materials & experimental systems

n/a	Involved in the study
☒	☐ Antibodies
☒	☐ Eukaryotic cell lines
☒	☐ Palaeontology and archaeology
☒	☐ Animals and other organisms
☒	☐ Clinical data
☒	☐ Dual use research of concern
☒	☐ Plants

Methods

n/a	Involved in the study
☒	☐ ChIP-seq
☒	☐ Flow cytometry
☒	☐ MRI-based neuroimaging
